# Formate dehydrogenase, ubiquinone, and cytochrome *bd*-I are required for peptidoglycan recognition protein-induced oxidative stress and killing in *Escherichia coli*

**DOI:** 10.1038/s41598-020-58302-1

**Published:** 2020-02-06

**Authors:** Des R. Kashyap, Dominik A. Kowalczyk, Yue Shan, Chun-Kai Yang, Dipika Gupta, Roman Dziarski

**Affiliations:** 1 Indiana University School of Medicine–Northwest, Gary, IN 46408 USA; 2 Antimicrobial Discovery Center, Northeastern University, Boston, MA 02115 USA; 3 Present Address: Department of Medicine, The University of Chicago, Chicago, 60637 USA

**Keywords:** Antimicrobial responses, Bacteriology

## Abstract

Mammalian Peptidoglycan Recognition Proteins (PGRPs) kill bacteria through induction of synergistic oxidative, thiol, and metal stress. PGRPs induce oxidative stress in bacteria through a block in the respiratory chain, which results in decreased respiration and incomplete reduction of oxygen (O_2_) to hydrogen peroxide (H_2_O_2_). In this study we identify the site of PGRP-induced generation of H_2_O_2_ in *Escherichia coli*. Tn-seq screening of *E. coli* Tn10 insertion library revealed that mutants in formate dehydrogenase (FDH) genes had the highest survival following PGRP treatment. Mutants lacking functional FDH-O had abolished PGRP-induced H_2_O_2_ production and the highest resistance to PGRP-induced killing, and formate enhanced PGRP-induced killing and H_2_O_2_ production in an FDH-dependent manner. Mutants in ubiquinone synthesis (but not menaquinone and demethylmenaquinone) and cytochrome *bd*-I (but not cytochromes *bo*_3_ and *bd*-II) also had completely abolished PGRP-induced H_2_O_2_ production and high resistance to PGRP-induced killing. Because electrons in the respiratory chain flow from dehydrogenases’ substrates through quinones and then cytochromes to O_2_, these results imply that the site of PGRP-induced incomplete reduction of O_2_ to H_2_O_2_ is downstream from dehydrogenases and ubiquinone at the level of cytochrome *bd*-I, which results in oxidative stress. These results reveal several essential steps in PGRP-induced bacterial killing.

## Introduction

Peptidoglycan Recognition Proteins (PGRPs) are evolutionarily conserved and function in antibacterial innate immunity^1,2^. Mammals have four PGRPs coded by *PGLYRP1-4* genes. PGLYRP1, PGLYRP3, and PGLYRP4 are directly bactericidal for both Gram-positive and Gram-negative bacteria^3–6^, whereas PGLYRP2 is an enzyme, peptidoglycan amidohydrolase^7,8^. All PGRPs have one or two conserved PGRP domains, which bind muramyl-peptide fragments of bacterial peptidoglycan^1,2^. Mammalian PGRPs also bind bacterial lipopolysaccharide (LPS) with a binding site located outside the peptidoglycan-binding groove^5,9^.

Bacterial killing by PGRPs requires binding of PGRP to peptidoglycan in Gram-positive bacteria or to the outer membrane in Gram-negative bacteria^10^. However, PGRPs do not enter the cytoplasm and exert bacterial killing from this extracellular site by simultaneously inducing three severe stress responses in bacteria: oxidative stress, thiol stress, and metal stress^10,11^. Simultaneous induction of all three stress responses is required for efficient PGRP-induced bacterial killing, because: (i) each stress response is required for PGRP-induced killing but individually each stress response is only bacteriostatic, but not bactericidal; and (ii) bacterial killing can be recapitulated by the simultaneous treatment of bacteria with paraquat (which induces H_2_O_2_ production), diamide (which depletes thiols), and metals (which increase intracellular metal concentrations)^11^.

Oxidative stress induced by PGRP in bacteria is due to increased production of hydrogen peroxide (H_2_O_2_) and hydroxyl radicals (HO^•^), which result in high induction of oxidative stress response genes, including OxyR and SoxR regulons in *Escherichia coli* and PerR regulon in *Bacillus subtilis*^10,11^. Production of H_2_O_2_ and HO^•^ is required for PGRP-induced killing, because: (i) under anaerobic conditions, when H_2_O_2_ and HO^•^ are not produced, PGRPs are only bacteriostatic, but not bactericidal^11^; (ii) inhibition of HO^•^ production by dipyridyl inhibits PGRP-induced bacterial killing^10^; (iii) Δ*recA E. coli* mutants (deficient in DNA repair) and Hpx^-^ *E. coli* and *B. subtilis* mutants (deficient in catalases and hydroxyperoxidases), known to be highly sensitive to H_2_O_2_ and HO^•^, are also more sensitive to PGRP-induced killing^11^; and (iv) *E. coli* mutants deficient in PGRP-induced H_2_O_2_ production are resistant to PGRP-induced killing^12^.

PGRP-induced thiol (disulfide) stress in both *E. coli* and *B. subtilis* is due to depletion of over 90% of intracellular thiols, which results in a great increase in the expression of thiol stress response genes, including many genes for chaperones and protein quality control^11^. PGRP-induced depletion of thiols is required for PGRP-induced killing, because thiourea (which protects against thiol depletion) diminishes PGRP-induced bacterial killing^10,11^. PGRP-induced metal stress is due to increases in intracellular free (labile) Zn^2+^ in *E. coli* and both Zn^2+^ and Cu^+^ in *B. subtilis*, which result in a great increase in the expression of metal efflux and metal detoxification genes^11^. PGRP-induced metal stress is also required for PGRP-induced killing of bacteria, because selective chelation of Zn^2+^ or Cu^+^ completely abolishes PGRP killing in both *E. coli* and *B. subtilis*^6,11^. However, PGRP-induced oxidative, thiol, and metal stress are mostly independent of each other^12^.

We recently discovered that bactericidal PGRP induces oxidative stress through a block in the respiratory chain, which results in a decrease in respiration and increased generation of H_2_O_2_, possibly due to premature diversion of electrons to O_2_^12^. This PGRP-induced production of H_2_O_2_ in *E. coli* depended on the increased supply of NADH from cAMP-Crp-controlled glycolysis and TCA cycle, and on oxidation of NADH to NAD^+^ by both NADH dehydrogenases, NDH-1 and NDH-2^12^. This conclusion was based on: (i) increased resistance to PGRP-induced killing and inability of PGRP to induce increased production of H_2_O_2_ in several deletion mutants for the components of this pathway, including Δ*nuo* (NDH-1 deficient), Δ*ndh* (NDH-2 deficient), several TCA-cycle enzymes (Δ*sucB*, Δ*sucD*, Δ*icd*, Δ*sdhD*, and Δ*lpdA*), and Δ*crp* and Δ*cyaA* (deficient in the cAMP-Crp regulator of TCA cycle and central carbon catabolism); (ii) correlated PGRP-induced increase in the expression of cAMP-Crp-controlled genes for central carbon catabolism and respiratory oxidoreductases; and (iii) correlated PGRP-induced increases in NADH, phosphoenolpyruvate, and cAMP in wild-type cells but not in the above PGRP-resistant mutants^12^. These results indicated that the PGRP-induced block in the respiratory chain and the site of generation of H_2_O_2_ occurred at or down-stream from NDH-1 and NDH-2. However, the exact location of this block and the site of production of H_2_O_2_ remained unknown.

The respiratory chain of *E. coli* is very complex and contains at least 15 dehydrogenases that can deliver electrons to 3 different quinones, and at least 14 terminal reductases that can deliver electrons to at least 8 different electron acceptors, including 3 cytochromes (*bo*_3_, *bd*-I, and *bd*-II), which deliver electrons to O_2_^13,14^. Therefore, in this study we used Tn-seq and targeted mutations to further identify which components of the respiratory chain participate in PGRP-induced killing of *E. coli* and to identify the site in the respiratory chain responsible for PGRP-induced H_2_O_2_ production and killing. Our results show that formate dehydrogenases (FDH), and especially FDH-O, are required for PGRP-induced production of H_2_O_2_ and killing in *E. coli*, in addition to the previously identified requirement for NDH-1 and NDH-2^12^. Our results further show that ubiquinone and cytochrome *bd*-I are also required for PGRP-induced killing, and that the site of PGRP-induced H_2_O_2_ production in the respiratory chain is downstream from FDH-O, NDH-1, NDH-2, and ubiquinone at the level of cytochrome *bd*-I.

## Results

### Tn-seq identifies formate dehydrogenase mutants with increased survival in PGRP-treated cultures

We used a highly saturated mini-Tn10 insertion library in *E. coli* MG1655 containing ~200,000 unique mutants, with each mutant containing one transposon randomly inserted into the chromosome^15^. The library was exposed for 3 hrs to BSA as a control or to a bactericidal concentration of PGRP that reduced the numbers of viable bacteria in 3 hrs by >99%, or to a sub-bactericidal (bacteriostatic) concentration of PGRP that reduced viable bacteria in 3 hrs by 50%. We selected 3 hrs for the assay to allow for the selection of mutants resistant to PGRP killing and the depletion of mutants more sensitive to PGRP killing. We used human recombinant PGLYRP4 as a representative bactericidal PGRP, as we have previously shown that all human bactericidal PGRPs had similar activity and mechanism of bacterial killing^4,6,10,11^. We used BSA-treated bacteria as a control (rather than the initial inoculum) to eliminate any possible differences in the frequencies of mutants that could be due to different growth rates of mutants during the 3-hr incubation period. We plated surviving bacteria for single colonies and identified mutated genes by Illumina Hi-seq. For each gene we calculated the survival index (SI), which reflects a change in the frequency of each Tn insertion mutant in PGRP-treated versus control (BSA) cultures. A neutral mutation with no effect on survival in the presence of PGRP has SI = 1, whereas SI > 1 denotes mutation that makes bacteria more resistant to PGRP (indicating that the product of the gene enhances PGRP killing), and SI < 1 denotes mutation that makes bacteria more sensitive to PGRP (indicating that the product of the gene protects bacteria from PGRP killing). In this study, we focused on the mutants with significantly increased SI to identify genes that participate in PGRP-induced killing.

**Figure 1:**
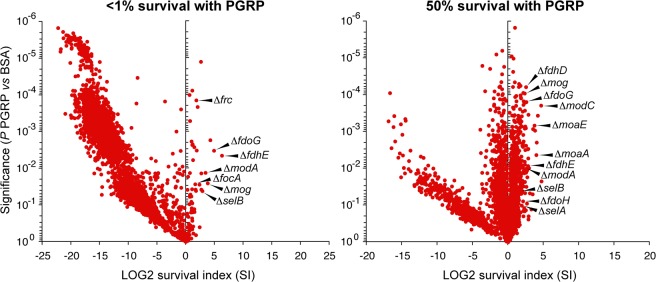
Tn-seq identifies association of formate dehydrogenase genes with PGRP-induced killing in *E. coli*. Tn10 insertion library was treated for 3 hrs with BSA as control or with PGRP at bactericidal (<1% survival) or sub-bactericidal (50% survival) concentration, and the survival index (SI) with PGRP, relative to BSA, for individual Tn-mutants was determined by Tn-seq. The results are means from 3 independent experiments (biological replicates) and each dot represents a single gene deletion mutant. The Tn insertion sites and the numbers of reads for the representative top FDH genes are shown in Supplementary Fig. S1. The complete Tn-seq results have been deposited in NCBI SRA with accession number PRJNA549505 (https://www.ncbi.nlm.nih.gov/sra/PRJNA549505).

Five out of the top six Tn-seq mutants with the most increased frequency in the Tn-seq library treated with bactericidal concentration of PGRP were in formate dehydrogenase (FDH) genes or in genes required for the assembly and activity of formate dehydrogenases (Figs. 1 and S1). The frequency of 11 Tn-seq mutants in formate dehydrogenases or in genes required for the assembly and activity of formate dehydrogenases was also significantly increased in bacteria treated with the bacteriostatic concentration of PGRP (Fig. 1).

*E. coli* has two respiratory formate dehydrogenases, FDH-O and FDH-N (coded by *fdoGHI* and *fdnGHI*, respectively), which transfer electrons from formate to the quinone pool in the respiratory chain. *E. coli* also has a third formate dehydrogenase, FDH-H (coded by *fdhF*), which is a subunit of formate hydrogenlyase, an enzyme complex that disproportionates formate to H_2_ and CO_2_^13,16,17^. All three FDH contain selenocysteine coordinated to molybdenum of molybdopterin guanine dinucleotide cofactor, which are both required for the FDH enzymatic activity. The mutants with increased frequency in PGRP-treated cultures were for the following genes (Fig. 1): *fdhE* (formate dehydrogenase formation protein required for the activity of FDH-O and FDH-N), *fdhD* (sulfurtransferase for molybdenum cofactor sulfuration required for the activity of FDH-O, FDH-N, and FDH-H), *fdoG* (catalytic α subunit of FDH-O), *fdoH* (β subunit of FDH-O), *modA* (molybdate ABC transporter periplasmic binding protein), *modC* (ATP-binding component of ABC transporter for high affinity uptake of molybdate), *mog* (molybdopterin adenylyltransferase), *moaA* (GTP 3′8′-cyclase in the synthesis of molybdopterin guanine dinucleotide), *moaE* (molybdopterin synthase catalytic subunit), *focA* (bidirectional formate channel), *selA* (selenocysteine synthase), *selB* (selenocysteinyl-tRNA-specific translation factor), and *frc* (formyl-CoA transferase).

### Formate dehydrogenase is required for PGRP-induced killing and H_2_O_2_ production

Increased frequency of Tn mutants in FDH genes and other genes required for the activity of FDH in PGRP-treated cultures suggested that FDH may participate in PGRP-induced killing of *E. coli*. To test this hypothesis and to identify which FDH participate in PGRP-induced killing, we constructed deletion mutants in key FDH genes or genes required for FDH activity and tested their sensitivity to PGRP-induced killing.

**Figure 2:**
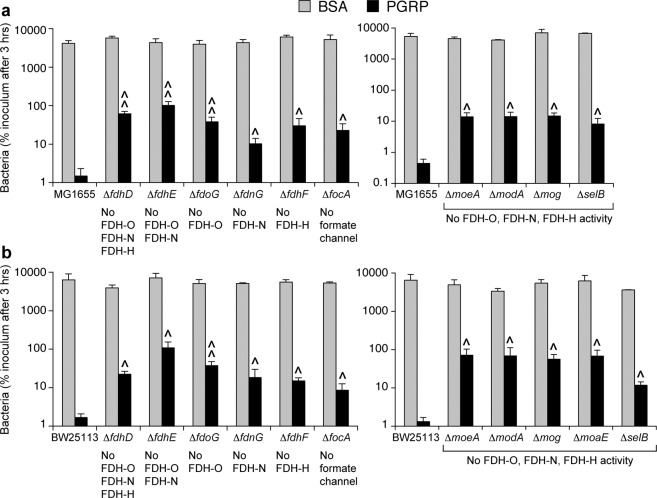
Deletion of genes for formate dehydrogenases (FDH) or genes required for the assembly and activity of FDH increases resistance of *E. coli* to PGRP-induced killing. Parental *E. coli* MG1655 (**a**) and BW25113 (**b**), or the indicated deletion mutants, were treated with BSA or PGRP for 3 hrs and the numbers of surviving bacteria were determined by colony counts. The results are expressed as percent of initial inoculum (100%) and are means ± SEM from 3 to 4 experiments (biological replicates); ^*P* < 0.05, ^^*P* < 0.001 mutant *vs* parental strain.

Mutants lacking FDH-O, FDH-N, and FDH-H (Δ*fdhD*), or FDH-O and FDH-N (Δ*fdhE*), or FDH-O (Δ*fdoG*) had significantly increased survival after 3-hr PGRP treatment compared with the parental MG1655 strain (with 51-, 66-, or 50-fold higher numbers of colonies, respectively) (Fig. 2a). Mutants lacking FDH-N (Δ*fdnG*), or FDH-H (Δ*fdhF*), or formate channel (Δ*focA*) also had significantly increased survival after 3-hr PGRP treatment, but much lower than the FDH-O mutants (7-, 18-, or 18-fold higher numbers of colonies than parental MG1655 strain, respectively) (Fig. 2a). Mutants lacking molybdopterin molybdenum transferase (Δ*moeA*), or molybdate transporter subunit (Δ*modA*), or molybdochelatase (Δ*mog*), or selenocysteinyl-tRNA-specific translation factor (Δ*selB*), which are all required for the activity of all FDH, also had significantly increased survival after 3-hr PGRP treatment (30-, 48-, 40-, or 26-fold higher numbers of colonies than parental MG1655 strain, respectively) (Fig. 2a).

We further confirmed the requirement for formate dehydrogenases in PGRP-induced killing by showing increased resistance to PGRP killing of Δ*fdhD*, Δ*fdhE*, Δ*fdoG*, Δ*fdnG*, Δ*fdhF*, Δ*focA*, Δ*moeA*, Δ*modA*, Δ*mog*, Δ*moaE*, and Δ*selB* deletion mutants in another strain of *E. coli*, BW25113 (Fig. 2b). These results indicate that FDH-O is required for PGRP-induced killing, and that FDH-N and FDH-H have a small, but still significant contribution.

We next hypothesized that formate may be a significant electron donor for the respiratory chain required for PGRP-induced H_2_O_2_ production and that formate dehydrogenases are required for PGRP-induced H_2_O_2_ production, because: (i) increased H_2_O_2_ production is required for PGRP-induced killing^11^; (ii) FDH transfer electrons from formate to the quinone pool in the respiratory chain^13,16^; and (iii) a block in the electron transfer through the respiratory chain was previously identified as a source of PGRP-induced H_2_O_2_^12^.

**Figure 3:**
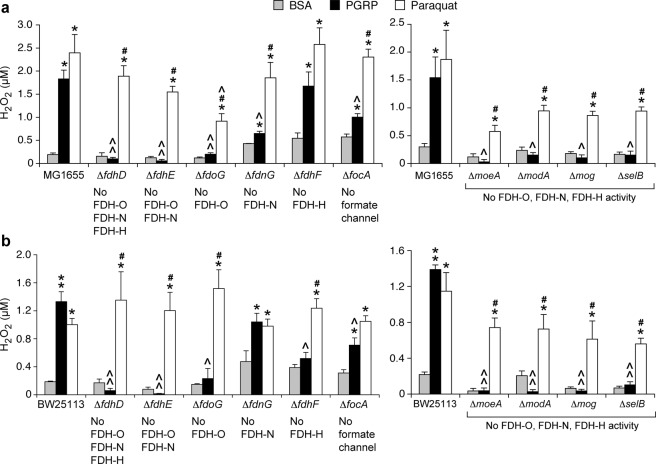
Deletion of FDH-O or genes required for FDH-O assembly and activity abolishes PGRP-induced production of H_2_O_2_ in *E. coli*. Parental strains MG1655 (**a**) and BW25113 (**b**), or the indicated deletion mutants, were treated with BSA, PGRP, or paraquat for 15 min and the total amounts of H_2_O_2_ were determined. The results are means ± SEM from 3 experiments (biological replicates); *P < 0.05, **P < 0.001 PGR*P* or paraquat *vs* BSA; ^#^*P* < 0.05 paraquat *vs* PGR*P*; ^*P* < 0.05, ^^*P* < 0.001 mutant *vs* parental strain.

Deleting FDH-O, FDH-N, and FDH-H (Δ*fdhD*), or FDH-O and FDH-N (Δ*fdhE*), or FDH-O (Δ*fdoG*) completely abolished PGRP-induced H_2_O_2_ production, whereas mutants lacking FDH-N (Δ*fdnG*) or formate channel (Δ*focA*) had still significant PGRP-induced H_2_O_2_ production, although lower than the parental MG1655 strain. Δ*fdhF* mutant lacking FDH-H (which does not directly contribute electrons to the respiratory chain) produced similar amounts of H_2_O_2_ in response to PGRP as the parental MG1655 strain (Fig. 3a). Mutants lacking molybdopterin molybdenum transferase (Δ*moeA*), or molybdate transporter subunit (Δ*modA*), or molybdochelatase (Δ*mog*), or selenocysteinyl-tRNA-specific translation factor (Δ*selB*) also did not produce H_2_O_2_ in response to PGRP.

We further confirmed the requirement for FDH-O in PGRP-induced H_2_O_2_ production by showing no increase in PGRP-induced H_2_O_2_ production in Δ*fdhD*, Δ*fdhE*, Δ*fdoG*, Δ*moeA*, Δ*modA*, Δ*mog*, Δ*moaE*, and Δ*selB* deletion mutants in *E. coli* BW25113 (Fig. 3b). Formate channel Δ*focA* BW25113 mutant behaved similarly to the Δ*focA* MG1655 mutant and showed significant, but lesser contribution, and FDH-N Δ*fdnG* and FDH-H Δ*fdhF* BW25113 mutants showed only smaller and variable contribution of FDH-N and FDH-H to PGRP-induced H_2_O_2_ production (Fig. 3b). Altogether, these results in MG1655 and BW25113 strains consistently indicate that FDH-O is required for PGRP-induced H_2_O_2_ production, whereas FDH-N and FDH-H have small and variable contributions.

The requirement for FDH-O in H_2_O_2_ production was selective for PGRP, because paraquat induced significant increases in H_2_O_2_ in both parental strains and all the mutants with non-functional FDH enzymes (Fig. 3). These results indicate that formate dehydrogenases are not required for generation of H_2_O_2_ by paraquat and are consistent with the known mechanism of H_2_O_2_ generation by paraquat, in which paraquat is reduced by respiratory NADH dehydrogenase to a radical cation, which then reacts with O_2_^18,19^.

**Figure 4:**
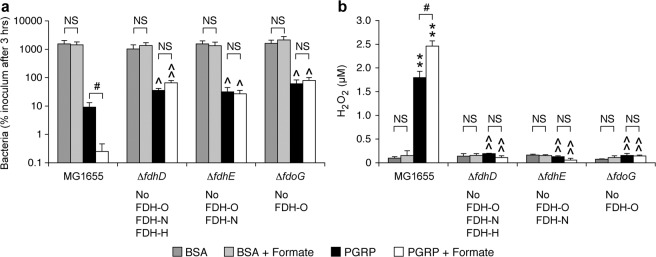
Exogenous formate enhances PGRP-induced killing and H_2_O_2_ production in *E. coli* and this enhancement is FDH-dependent. 0.25 mM sodium formate was added as indicated to the cultures of the parental strain MG1655 or FDH deletion mutants that were treated with BSA or PGRP and assayed for viability by colony counts after 3 hrs (**a**) or total amounts of H_2_O_2_ after 15 min (**b**) of incubation. The results are means ± SEM from 3 experiments (biological replicates); *P < 0.05, **P < 0.001 PGR*P vs* BSA; ^*P* < 0.05, ^^*P* < 0.001 mutant *vs* parental strain; ^#^*P* < 0.05 without *vs* with formate; NS *P* > 0.05.

To further determine the role of FDH in PGRP-induced killing and H_2_O_2_ production, we tested the hypothesis that adding exogenous formate should enhance PGRP-induced killing and H_2_O_2_ production. As predicted, adding 0.25 mM sodium formate significantly increased PGRP-induced killing and H_2_O_2_ production in *E. coli* MG1655 parental strain, but not in Δ*fdhD*, Δ*fdhE*, Δ*fdoG* mutants, showing that this effect of formate was dependent on FDH-O (Fig. 4). Exogenous formate had no effect on the bacterial viability and H_2_O_2_ concentrations in BSA-treated cultures showing the selectivity of the effect of formate in PGRP-treated cells.

To verify the presence of FDH-O, FDH-N, and FDH-H, we compared the expression of these FDH and other genes required for FDH assembly and activity in control and PGRP-treated cells. We analyzed whole genome expression arrays in BSA- (control) and PGRP-treated *E. coli*, which we previously deposited in NCBI GEO (accession number GSE44211) and at that time analyzed for the oxidative, thiol, and metal stress response genes^11^, but not for FDH-related genes. Control (BSA-treated) cells had high expression of *fdhD* and *fdhE* (required for the activity of FDH-O, FDH-N, and FDH-H, or FDH-O and FDH-N, respectively), *fdoG*, *fdoH*, and *fdoI* (coding for FDH-O subunits), and many genes required for the synthesis of the molybdopterin guanine dinucleotide cofactor and selenocysteine (required for the activity of all three FDH enzymes) (Supplementary Fig. S2). Control cells had low expression of FDH-N (*fdnG*, *fdnH*, and *fdnI*) and FDH-H (*fdhF*) genes. PGRP treatment significantly increased the expression of *fdoG*, *fdoH*, *fdoI*, *fdnH*, and *fdnI*, and also of several molybdopterin and selenocysteine synthesis genes (Supplementary Fig. S2). These results are consistent with aerobic expression of FDH-O^16^ and our results showing its role in PGRP-induced killing and H_2_O_2_ production, and also show that expression of FDH-N can be induced aerobically in PGRP-treated cells, consistent with partial contribution of FDH-N to PGRP-induced killing.

### Ubiquinone is required for PGRP-induced killing and H_2_O_2_ production

FDH-O and FDH-N transfer electrons from formate to the quinone pool in the cytoplasmic membrane, and these electrons are then transferred from quinones to the cytochromes, which in the presence of oxygen are the terminal oxygen reductases that reduce O_2_ to H_2_O. The requirement for FDH-O in PGRP-induced H_2_O_2_ production (Fig. 3) and simultaneous PGRP-induced decrease in O_2_ consumption^12^ indicated a block in the respiratory chain and either premature diversion of electrons from the respiratory chain or its malfunction, leading to incomplete reduction of O_2_ to H_2_O_2_. This PGRP-induced block in electron transfer and incomplete reduction of O_2_ to H_2_O_2_ could occur at the level of dehydrogenases (FDH and/or NDH), quinones, or cytochromes. If the diversion of electrons occurred from the dehydrogenases and not from quinones (located downstream in the electron transfer chain), deleting quinones should *not* abolish the PGRP-induced H_2_O_2_ production. But if the diversion of electrons occurred at the level of quinones or downstream from quinones, deleting quinones should abolish or decrease PGRP-induced H_2_O_2_ production. To discriminate between these possibilities and to identify which quinones (if any) are involved in PGRP-induced H_2_O_2_ production and PGRP-induced killing, we next tested the PGRP-induced killing and H_2_O_2_ production in quinone deletion mutants.

**Figure 5:**
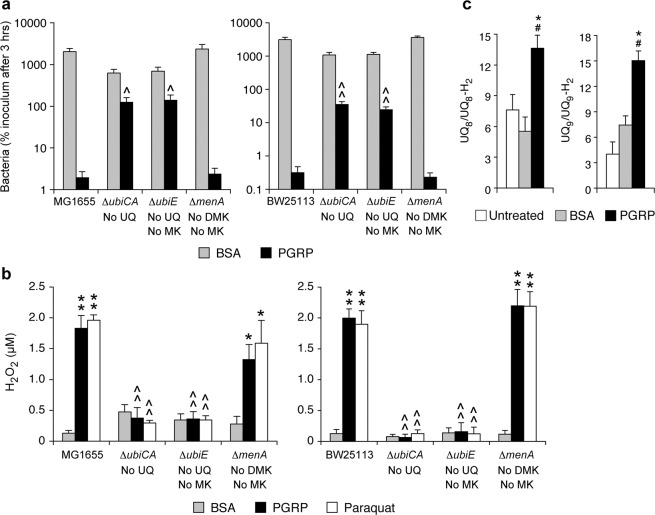
Deletion of ubiquinone (UQ) synthesis genes increases resistance of *E. coli* to PGRP-induced killing (**a**) and decreases PGRP-induced H_2_O_2_ production (**b**); and the ratio of oxidized to reduced UQ is increased in PGRP-treated compared with BSA-treated *E. coli* (**c**). Parental *E. coli* and deletion mutants were treated with BSA, PGRP, or paraquat, as indicated. (**a**) The numbers of surviving bacteria at 3 hrs were determined by colony counts and expressed as percent of initial inoculum (100%). (**b**) The total amounts of H_2_O_2_ at 15 min were determined. The results are means ± SEM from 4 (**a**) or 6 (**b**) experiments (biological replicates); *P < 0.05, **P < 0.001, *P*GRP or paraquat *vs* BSA; ^*P* < 0.05, ^^*P* < 0.001, mutant *vs* parental strain. (**c**) The amounts of oxidized and reduced UQ were determined in MG1655 *E. coli* at time 0 (untreated), and after 15 min of incubation with BSA (control) or PGRP. The results are mean ratios ± SEM of UQ_8_/UQ_8_-H_2_ and UQ_9_/UQ_9_-H_2_ from 4 experiments (biological replicates); ^#^*P* < 0.05 PGRP *vs* untreated, *P < 0.05 PGRP *vs* BSA (paired *t*-test). The total amounts of UQ + UQ-H_2_ in untreated, BSA-treated, and PGRP-treated cultures were: 107 ± 5, 161 ± 19, and 149 ± 15 ng/culture for UQ_8_, and 1.7 ± 0.05, 2.0 ± 0.4, and 2.2 ± 0.2 ng/culture for UQ_9_, respectively (means ± SEM), with no significant differences between BSA- and PGRP-treated groups.

*E. coli* has 3 quinones: ubiquinone (UQ), menaquinone (MK), and demethylmenaquinone (DMK), which differ in their redox potential and thus affinity for electrons^20^. We constructed mutants deficient in the synthesis of UQ, MK, and DMK in both MG1655 and BW25113 strains and tested their sensitivity to PGRP-induced killing and H_2_O_2_ production. Mutants deficient in the synthesis of UQ (Δ*ubiCA*) or UQ and MK (Δ*ubiE*) had significantly increased survival after 3-hr PGRP treatment (with 65- and 72-fold higher numbers of colonies than in the parental MG1655 strain, and 111- and 77-fold higher in BW25113 strain, respectively) (Fig. 5a). By contrast, mutants deficient in the synthesis of both MK and DMK (Δ*menA*) had unchanged survival, compared with the parental MG1655 and BW25113 strains (Fig. 5a). Thus, UQ, but not MK and DMK, is required for PGRP-induced killing.

In mutants lacking UQ (Δ*ubiCA*) or UQ and MK (Δ*ubiE*) PGRP did not induce any H_2_O_2_ production, whereas mutants lacking both MK and DMK (Δ*menA*) had significant PGRP-induced H_2_O_2_ production that did not significantly differ from H_2_O_2_ production in the parental strains (Fig. 5b). Mutants lacking UQ (but not MK and DMK) also had similarly abolished H_2_O_2_ production in response to paraquat, which shows that UQ is required for paraquat-generated H_2_O_2_ production, consistent with the proposed ability of quinones to participate in the redox cycling of paraquat^21^. Similar results were obtained in mutants independently generated in both MG1655 and BW25113 strains, validating that these effects were indeed due to the deletion of these quinone synthesis genes. These results indicate that UQ (but not MK and DMK) is required for PGRP-induced H_2_O_2_ production. Thus, the site of PGRP-induced H_2_O_2_ production in the respiratory electron transport chain is at the level of UQ or downstream from UQ.

If ubiquinol (UQ-H_2_) is the source of electrons for H_2_O_2_ production in PGRP-treated cells, the outcome of increased transfer of electrons to O_2_ should be an increase in oxidized UQ at the time of peak H_2_O_2_ production. To test this hypothesis we measured the amounts of oxidized and reduced quinones after 15 min of PGRP treatment, which is the peak of PGRP-induced H_2_O_2_ production^12^. We focused on UQ, because UQ, but not MK and DMK, was required for PGRP-induced H_2_O_2_ production and killing (Fig. 5a,b), and because UQ is the main quinone in aerobically growing *E. coli*^20,22^. Indeed, we detected a total of approximately 140 ng UQ_8_, 2.0 ng UQ_9_, 3.3 ng MK_8_, and <0.1 ng DMK_8_ (below the level of detection) per culture (oxidized plus reduced forms).

A 15-min treatment with PGRP resulted in significant increases in both UQ_8_/UQ_8_-H_2_ and UQ_9_/UQ_9_-H_2_ (oxidized/reduced) ratios, compared with untreated and BSA-treated cells, indicating a significant shift from reduced UQ-H_2_ to oxidized UQ in PGRP-treated cells (Fig. 5c). By contrast, these ratios were similar in untreated and BSA-treated cells. These results are consistent with our hypothesis of increased loss of electrons from reduced UQ-H_2_ resulting in an increase in the oxidized UQ/reduced UQ-H_2_ ratio in PGRP-treated cells at the time of the maximum H_2_O_2_ production. These electrons could be diverted to O_2_ directly from UQ-H_2_ to generate H_2_O_2_, or transferred to cytochromes, or both.

To further verify the presence of quinones, we compared the expression of quinone synthesis genes in control and PGRP-treated cells, by analyzing whole genome expression arrays, which we previously deposited in NCBI GEO (accession number GSE44211)^11^, but did not analyze for quinone synthesis. Control (BSA-treated) cells had high expression of genes for the synthesis of UQ (*ubiA*, *ubiC*, and *ubiE*), and expression of *ubiA* and *ubiC* (required for the synthesis of UQ) was further increased by PGRP treatment (Supplementary Fig. S3a). Expression of *menA*, required for the synthesis of both MK and DMK, was lower and significantly decreased by PGRP treatment. These results are consistent with the dominant role of UQ in PGRP-induced killing and H_2_O_2_ production.

### Cytochrome *bd*-I is required for PGRP-induced killing and H_2_O_2_ production

Our results showing the requirement for UQ in PGRP-induced killing and H_2_O_2_ production motivated us to determine the role of cytochromes in PGRP-induced killing and H_2_O_2_ production, because in the respiratory chain UQ-H_2_ transfers electrons to cytochromes. Thus, we next tested whether the PGRP-induced block in the respiratory chain and the site of incomplete reduction of O_2_ to H_2_O_2_ occurred down-stream from the quinones, i.e., at the cytochromes. *E. coli* has 3 terminal quinol/oxygen oxidoreductases: cytochromes *bo*_3_, *bd*-I, and *bd*-II^13,14^. We constructed mutants deficient in each cytochrome in *E. coli* MG1655 and tested the sensitivity of cytochrome mutants in both MG1655 and BW25113 strains to PGRP-induced killing and H_2_O_2_ production.

**Figure 6:**
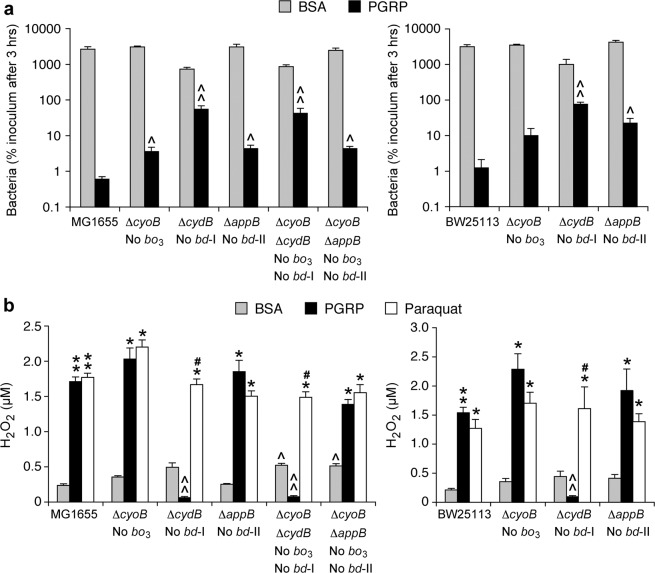
Deletion of cytochrome *bd*-I increases resistance to PGRP-induced killing and abolishes PGRP-induced H_2_O_2_ production. Parental *E. coli* or deletion mutants were treated as indicated. (**a**) The numbers of surviving bacteria after 3 hrs were determined by colony counts and expressed as percent of initial inoculum (100%). (**b**) The total amounts of H_2_O_2_ after 15 min were measured. The results are means ± SEM from 3 or 4 experiments (biological replicates); *P < 0.05, **P < 0.001, *P*GRP or paraquat *vs* BSA; ^#^*P* < 0.05 paraquat *vs* PGR*P*; ^*P* < 0.05, ^^*P* < 0.001, mutant *vs* parental strain.

Mutants lacking cytochrome *bd*-I (Δ*cydB*) had significantly increased survival after 3-hr PGRP treatment (with 92- and 61-fold higher numbers of colonies than in the parental MG1655 and BW25113 strains, respectively) (Fig. 6a). Single deletion mutants lacking cytochrome *bo*_3_ (Δ*cyoB*) or cytochrome *bd*-II (Δ*appB*) had significantly lower survival than the Δ*cydB* mutant, but they still had somewhat higher survival than the parental strains (with 6- and 7-, or 8- and 18-fold higher numbers of colonies than in the parental MG1655 and BW25113 strains, respectively). Double deletion Δ*cyoB*Δ*cydB* and Δ*cyoB*Δ*appB* mutants also had significantly increased survival after 3-hr PGRP treatment (with 71- or 7-fold higher numbers of colonies than in the parental MG1655 strain), similar to the survival of the single Δ*cydB* and Δ*cyoB* mutants, respectively. These results indicate that cytochrome *bd*-I is required for PGRP-induced killing, and that cytochromes *bo*_3_ and *bd*-II play a small role.

PGRP-induced H_2_O_2_ production was completely abolished in both MG1655 and BW25113 mutants lacking cytochrome *bd*-I (Δ*cydB*), and also in double deletion Δ*cyoB*Δ*cydB* mutant, compared with the parental strains (Fig. 6b). By contrast, PGRP-induced H_2_O_2_ production in both MG1655 and BW25113 mutants lacking cytochrome *bo*_3_ (Δ*cyoB*) or cytochrome *bd*-II (Δ*appB*), or in a double deletion mutant lacking both cytochromes *bo*_3_ and *bd*-II (Δ*cyoB*Δ*appB*), were similar to the H_2_O_2_ production in the parental strains. These results show that cytochrome *bd*-I is required for PGRP-induced H_2_O_2_ production and that cytochromes *bo*_3_ and *bd*-II are not required. These results imply that the primary site of PGRP-induced production of H_2_O_2_ is cytochrome *bd*-I.

The requirement for cytochrome *bd*-I in H_2_O_2_ production was selective for PGRP, because paraquat induced significant increases in H_2_O_2_ in both parental strains and all the cytochrome mutants, including mutants lacking cytochrome *bd*-I (Fig. 6b). These results indicate no selective requirement for any of the *E. coli* cytochromes for paraquat-induced H_2_O_2_ production (in contrast to PGRP) and are consistent with the known direct reduction of O_2_ by the paraquat radical cation^18,19^.

Both double deletion mutants (Δ*cyoB*Δ*cydB* and Δ*cyoB*Δ*appB*) had higher H_2_O_2_ production in control (BSA-treated) cultures than the parental strain (Fig. 6b), which is consistent with the previous report for the untreated Δ*cyo*Δ*cyd* mutant^23^. Because PGRP-induced H_2_O_2_ production was completely abolished in Δ*cydB* and Δ*cyoB*Δ*cydB* mutants, these results indicate that the main source of PGRP-induced H_2_O_2_ is different from the sources of the low-level H_2_O_2_ produced in control cells growing without PGRP.

To verify the presence of cytochromes, we compared the expression of cytochrome genes in control and PGRP-treated cells, by analyzing our whole genome expression arrays (NCBI GEO accession number GSE44211)^11^. Control (BSA-treated) cells had high expression of cytochrome *bo*_3_ *cyoA-D* genes, most of which were further increased by PGRP treatment, and very high expression of cytochrome *bd*-I *cydABX* genes. However, the expression of cytochrome *bd*-I *cydA* gene was severely decreased by PGRP treatment (Supplementary Fig. S3b). Cytochrome *bd*-II *appB* and *appC* genes had very low expression, which was not changed by PGRP treatment. These results are consistent with the role of cytochrome *bd*-I in PGRP-induced killing and H_2_O_2_ production and suggest that maybe PGRP-treated cells decrease cytochrome *bd*-I expression as a defense mechanism to limit its contribution to PGRP-induced H_2_O_2_ production.

### Role of membrane depolarization

**Figure 7:**
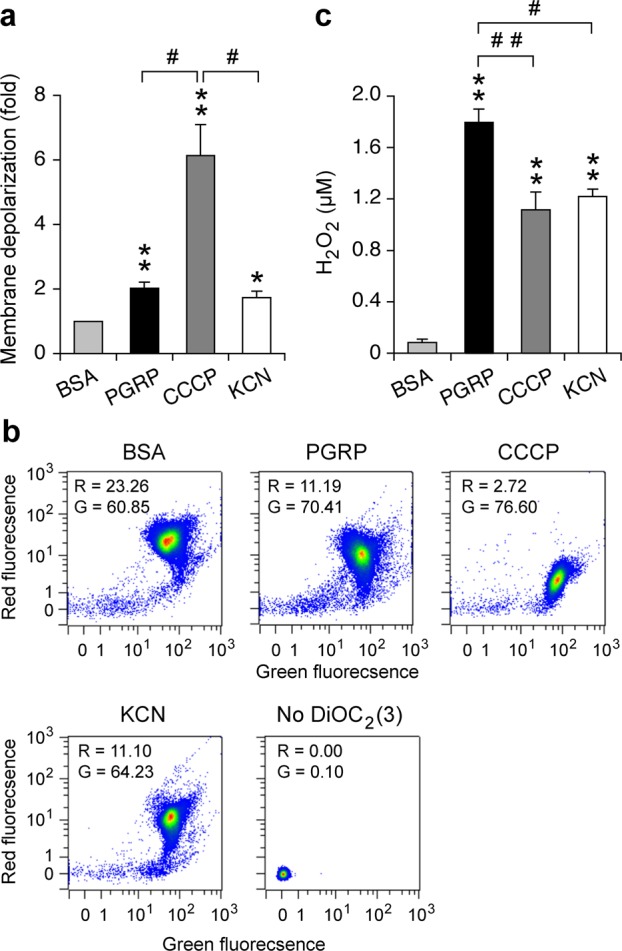
Comparison of membrane depolarization and H_2_O_2_ production. *E. coli* were treated with BSA, PGRP, CCCP, or KCN as indicated for 15 min. (**a**) Membrane depolarization was measured by flow cytometry with membrane potential sensitive dye DiOC_2_(3) and the results are the ratios of mean red fluorescence intensity in BSA/treated cells shown as means ± SEM from 6 experiments. (**b**) Representative dot plots with mean red (R) and green (G) fluorescence intensities. (**c**) Total amounts of H_2_O_2_ are shown as means ± SEM from 8 experiments. *P < 0.05, **P < 0.001, *P*GRP, CCCP, or KCN *vs* BSA; ^#^*P* < 0.05, ^##^*P* < 0.001 as indicated; all other differences were not significant (*P* > 0.05).

We next considered the possibility that PGRP induces membrane depolarization in *E. coli* because membrane depolarization could be either the cause or the consequence of respiratory chain malfunction, oxidative stress, and decline in respiration, and because we previously showed that PGRP treatment results in membrane depolarization in *B. subtilis*^10^. Treatment of *E. coli* with PGRP for 15 min (the time of maximum PGRP-induced H_2_O_2_ production^12^) resulted in significant, but low-level membrane depolarization, similar to membrane depolarization induced by 100 µM KCN, a cytochrome *bo*_3_ inhibitor^14^, as measured by a decrease in red fluorescence of membrane potential sensitive probe DiOC_2_(3) (Fig. 7a,b). KCN-induced membrane depolarization is consistent with inhibition of cytochrome *bo*_3_ and its proton-pumping ability^13,14^. CCCP (carbonyl cyanide 3-chlorophenylhydrazone), a proton ionophore that dissipates both membrane potential and proton gradient, induced strong membrane depolarization (as expected), 3-fold (significantly) higher than PGRP and KCN (Fig. 7a,b).

We next tested whether membrane depolarization with CCCP or cytochrome inhibition with KCN would induce H_2_O_2_ production and whether the amount of H_2_O_2_ would correlate with the extent of membrane depolarization. Both CCCP and KCN induced similar and significant increases in H_2_O_2_ production compared with BSA-treated bacteria, but the amounts of CCCP- and KCN-induced H_2_O_2_ were significantly lower than in PGRP-treated bacteria (Fig. 7c). These results reveal that membrane depolarization with CCCP or cytochrome *bo*_3_ inhibition with KCN induce significant H_2_O_2_ production, but also show that there is no correlation between the extent of membrane depolarization and the amount of H_2_O_2_ produced, as PGRP induced low-level membrane depolarization and high amount of H_2_O_2_, whereas CCCP induced much higher level of membrane depolarization but lower amount of H_2_O_2_ than PGRP.

## Discussion

**Figure 8:**
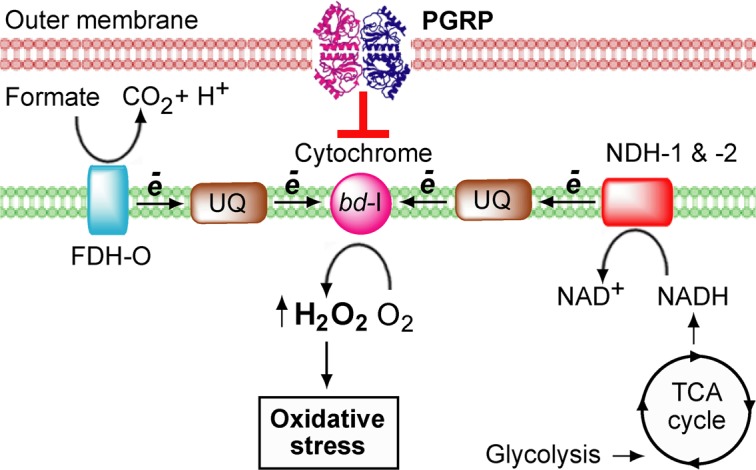
Proposed events in PGRP-induced oxidative stress in *E. coli*. Electrons from FDH-O, NDH-1, and NDH-2 substrates flow through UQ to cytochrome *bd*-I, which results in partial reduction of O_2_ to H_2_O_2_, likely at the level of cytochrome *bd*-I due to incomplete electron transfer from UQ-H_2_ to cytochrome *bd*-I or malfunction of cytochrome *bd*-I. The amount of H_2_O_2_ exceeds the maximum level that can be detoxified by the cell and induces oxidative stress.

*E. coli* has a complex branched respiratory chain, which allows the bacterium to quickly adapt to changes in the oxygen supply and other environmental conditions^13^. However, these multiple and essential functions of *E. coli* respiratory chain are also a target for human bactericidal innate immunity proteins, PGRPs, which induce a block in *E. coli* respiratory chain^12^. The results of this study show that PGRP-induced H_2_O_2_ production and bacterial killing require FDH-O, UQ, and cytochrome *bd*-I, in addition to the previously shown requirement for NDH-1 and NDH-2^12^. PGRP-treated *E. coli* up-regulates its metabolism by increasing cAMP-Crp-controlled TCA cycle and supply of NADH, by increasing NADH oxidation by NDH-1 and NDH-2^12^, and also by increasing oxidation of formate by FDH-O, perhaps in an attempt to overcome this block in the respiratory chain by increasing influx of electrons. The central and critical components of this proposed model are UQ and cytochrome *bd*-I, because increased function of all three dehydrogenases (FDH-O, NDH-1, and NDH-2) supplies electrons to UQ and reduces it to ubiquinol (UQ-H_2_), which then transfers electrons to cytochromes (Fig. 8).

We propose that cytochrome *bd*-I is the site of PGRP-induced H_2_O_2_ production and not the dehydrogenases (FDH and NDH) and UQ, because deleting UQ (downstream from FDH and NDH) or cytochrome *bd*-I (downstream from UQ) completely abolished PGRP-induced H_2_O_2_ production. If FDH, NDH or UQ were the sites of H_2_O_2_ production due to electron backup, deleting downstream components of the respiratory chain (UQ or cytochrome *bd*-I) would have had no effect or would have enhanced H_2_O_2_ production. Thus, our data showing that deleting dehydrogenases^12^, or UQ, or cytochrome *bd*-I all abolished PGRP-induced H_2_O_2_ production suggest that electrons are being transferred all the way down to cytochrome *bd*-I and that incomplete reduction of O_2_ to H_2_O_2_ is at the level of cytochrome *bd*-I, possibly due to incomplete electron transfer from UQ-H_2_ to cytochrome *bd*-I or malfunction of cytochrome *bd*-I (Fig. 8).

Consistent with this proposed model, there was a significant increase in UQ/UQ-H_2_ ratio at the peak of PGRP-induced H_2_O_2_ production (15 min), suggesting that the increased electron transfer from UQ-H_2_ to cytochrome *bd*-I at that time had already occurred and resulted in UQ-H_2_ oxidation to UQ. This high UQ/UQ-H_2_ ratio is maintained, because in PGRP-treated cells there is only a short-lived spike in the metabolism (5 to 10 min) followed by a decrease^12^, which limits further supply of electrons to UQ.

Decreasing O_2_ consumption at the time of PGRP-induced H_2_O_2_ production^12^ suggests that this H_2_O_2_ production is not a small byproduct of the usual O_2_ reduction to H_2_O by the cytochromes, because this would require a very large PGRP-induced increase in O_2_ consumption to produce such a large amount of H_2_O_2_ (as a byproduct). Therefore, the diminished O_2_ consumption^12^ concomitant with a high increase in H_2_O_2_ production can be best explained by incomplete reduction of the majority of O_2_ to H_2_O_2_ and thus even a decreasing O_2_ consumption could still produce the amounts of H_2_O_2_ that we detected. With continued PGRP treatment the function of the respiratory chain progressively deteriorates, eventually leading to almost complete loss of O_2_ consumption and a decline in PGRP-induced H_2_O_2_ production at 30 min^12^, which coincides with the cessation of all biosynthetic reactions in the cell^10^.

Our proposed model is energetically favorable for efficient transfer of electrons (exergonic)^13^. CO_2_/formate coupled to FDH has the lowest midpoint potential (*E*_o_′ = −432 mV with donor/UQ Δ*E*_o_′ = 545 mV), followed by NAD^+^/NADH coupled to NDH-1 and NDH-2 (*E*_o_′ = −320 mV with donor/UQ Δ*E*_o_′ = 433 mV), O_2_/H_2_O_2_ coupled to cytochrome *bd*-I (*E*_o_′ = 280 mV with UQ-H_2_/acceptor Δ*E*_o_′ = 167 mV), and O_2_/H_2_O coupled to cytochrome *bd*-I (*E*_o_′ = 820 mV with UQ-H_2_/acceptor Δ*E*_o_′ = 707 mV)^13,14,24^. Cytochrome *bd*-I has three hemes, *b*_558_, *b*_595_, and *d*, with *E*_o_′ = 176 mV, 168 mV, and 258 mV, respectively, that can accept electrons^14,24^. *E. coli* UQ/UQ-H_2_ *E*_o_′ = 113 mV and thus it is energetically favorable for cytochrome *bd*-I to accept electrons from UQ-H_2_. Because heme *d* is the main O_2_-binding and reducing site in cytochrome *bd*-I^14,24^, reducing O_2_ to H_2_O_2_ (O_2_/H_2_O_2_ *E*_o_′ = 280 mV) at heme *d* is still energetically possible with Δ*E*_o_′ = 22 mV, although less favorable than reducing O_2_ to H_2_O (Δ*E*_o_′ = 562 mV). Cytochrome *bo*_3_ has two hemes, *b* and *o*_3_, and Cu_B_ that accept electrons with *E*_o_′ = 280 mV, 280 mV, and 370 mV, respectively (with *o*_3_/Cu_B_ donating electrons to O_2_)^14^, and thus it is not energetically favorable for cytochrome *bo*_3_ to reduce O_2_ to H_2_O_2_, which may be the reason why deleting cytochrome *bo*_3_ has no effect on PGRP-induced H_2_O_2_ production.

How do PGRPs induce the respiratory chain malfunction that leads to H_2_O_2_ production? PGRPs have a peptidoglycan-binding groove specific for disaccharide-peptide fragments of peptidoglycan. In Gram-positive bacteria PGRPs selectively bind to the separation sites of the newly formed daughter cells, created by dedicated peptidoglycan-lytic endopeptidases, which separate daughter cells after cell division^10^. In Gram-negative bacteria, PGRPs bind to the entire outer membrane^10^ through a separate binding site specific for LPS^5,9^. This binding to the outer membrane is required for PGRP killing, because exogenous LPS blocks PGRP binding and PGRP killing of *E. coli*^10^. PGRPs do not enter the cytoplasm^10^ and induce the respiratory chain malfunction from the extracellular site, but it is not known whether after binding to the outer membrane they translocate to peptidoglycan in the periplasmic space and/or to the cytoplasmic membrane. Therefore, it is not known whether the effects of PGRPs on the respiratory chain are indirect (more likely) or direct.

We considered that PGRP-induced membrane damage or depolarization could be the cause of the respiratory chain malfunction and H_2_O_2_ production. However, cytoplasmic membrane is not permeabilized in PGRP-treated *E. coli* for up to 6 hrs^6^ and PGRP induced only low-level membrane depolarization. Moreover, there was no correlation between the extent of membrane depolarization and the amount of H_2_O_2_ produced, i.e., PGRP induced low membrane depolarization and high H_2_O_2_ production, whereas CCCP induced high membrane depolarization and lower H_2_O_2_ production (Fig. 7). These results are consistent with our previous analysis of gene expression^11^, which showed high induction of oxidative stress response genes in PGRP-treated *E. coli*, such as *oxyS*, *ahpF*, *katG*, *soxS*, and *soxR* (indicating high production of H_2_O_2_ and O_2_^−^), but no increase in CCCP-treated cells in the expression in *oxyS*, *ahpF*, and *katG*, and moderate increase in the expression of *soxS* and *soxR* (indicating production of some O_2_^−^ but no or low amount of H_2_O_2_) and thus showing very limited oxidative stress response to CCCP. Altogether, these results suggest that PGRP-induced membrane depolarization may be the consequence of the decreased respiration rather than the primary cause of the respiratory chain malfunction and H_2_O_2_ production. However, other possibilities cannot be excluded, such as membrane depolarization as the initial event, or other effects of PGRP on the cytoplasmic membrane that interfere with the respiratory chain.

The subsequent events that follow PGRP interaction with bacteria and production of H_2_O_2_ and contribute to the eventual bacterial death include: (i) depletion of cellular thiols (peaks in 30 min); (ii) generation of HO^•^ from H_2_O_2_, which then damage bacterial DNA, membrane, and proteins (30 min–2 hrs); (iii) increase in intracellular labile Zn^2+^ (peaks in 1 hr); and (iv) cessation of all biosynthetic reactions (30 min–2 hrs)^10–12^.

Our results also identify a new function for FDH-O. FDH-O and FDH-N are transmembrane dehydrogenases (formate-quinone oxidoreductases) with their catalytic sites facing the periplasmic space. They oxidize periplasmic formate to CO_2_ and reduce membrane quinones to quinols, which then transfer electrons to the terminal respiratory chain quinol oxidoreductases. Whereas much is known about the structure and function of FDH-N in anaerobic respiration on nitrite and nitrate^13,16,17^, very little is known about the function of aerobically expressed FDH-O^16,17^. The requirement for FDH-O in PGRP-induced bacterial killing and H_2_O_2_ production clearly show that FDH-O is active in aerobically growing *E. coli* and is an integral and important component of the aerobic respiratory chain and a major contributor of electrons to the quinone pool. Our results confirm abundant aerobic expression of FDH-O. The importance of FDH-O is also highlighted by recent results of Hughes *et al*.^25^ showing the ability of *Enterobacteria* to use formate oxidation by FDH-O and FDH-N to shuttle electrons to the respiratory chain cytochrome *bd*-I when oxygen becomes available. Hughes *et al*. results also suggests that O_2_ is the final electron acceptor for formate oxidation by both FDH-N and FDH-O (which is consistent with our results) and show that this mechanism gives *Enterobacteria* a competitive advantage in the inflamed gut, where oxygen is supplied by blood. Our results suggest that PGRPs can exploit this mechanism to kill bacteria.

Our results show that multiple dehydrogenases – FDH-O (this study) and NDH-1 and NDH-2^12^ – are all required for PGRP-induced killing and H_2_O_2_ production. Moreover, elimination of even a single dehydrogenase results in a significantly increased resistance to PGRP-induced killing and in a drastic loss of PGRP-induced H_2_O_2_ production, rather than in a small incremental effect of each deletion. One possible explanation for this finding could be a threshold effect, in which simultaneous action of all the dehydrogenases is required to produce the amount of H_2_O_2_ that exceeds the maximum level that the cell can detoxify, and deletion of any single dehydrogenase decreases the amount of H_2_O_2_ below this threshold level.

Another possible explanation could be the formation of supercomplexes by the respiratory dehydrogenases and cytochromes, in which the function of each component is dependent on the presence and/or function of the other components in the supercomplex. Formation of such supercomplexes is well accepted in mitochondria^26,27^. In *E. coli* formation of three supercomplexes was reported: FDH-O:cytochrome *bo*_3_:cytochrome *bd*-I supercomplex, NDH-1:NDH-2 supercomplex, and SDH:cytochrome *bd*-II supercomplex, with each component of the complex required for the assembly and full enzymatic activity of the supercomplex^26,28–30^. Super-resolution microscopy and quantification revealed the presence of complexes of respiratory chain enzymes in *E. coli* membrane, but did not show co-localization of NDH-1, cytochrome *bo*_3_, cytochrome *bd*-I, SDH, and F_o_F_1_ATPase in these complexes^27^. These results do not support the presence of the above-mentioned supercomplexes and make this possibility a less likely explanation of our results, although co-localization of FDH-O and NDH-2 with other respiratory enzymes was not studied in this report^27^ and is still a possibility.

Cytochromes usually serve as electron sinks and under normal growth conditions protect bacteria from inadvertent production of H_2_O_2_ and oxidative stress, because their high affinity for O_2_ allows them to efficiently transfer 4 electrons to O_2_ for its complete reduction to H_2_O^13,14,31^. However, our results suggest that in PGRP-treated cells cytochrome *bd*-I malfunctions, which results in an incomplete 2-electron reduction of O_2_ to H_2_O_2_. Thus, the pathways and the final electron donors (cytochromes) for PGRP-induced H_2_O_2_ are likely different from the sources of the low-level H_2_O_2_ produced in control cells growing without PGRP, because deleting NDH-1 and NDH-2, or UQ, or cytochromes *bo*_3_ and *bd*-I increase the basal levels of H_2_O_2_ production in cells growing in a medium^23^, whereas, these deletions in PGRP-treated cells resulted in a decrease of PGRP-induced H_2_O_2_ production (ref. ^12^ and Figs. 5 and 6).

Purified cytochrome *bd*-I has peroxidase activity and can reduce H_2_O_2_ to H_2_O using quinol as an electron donor^32^. It is not known how this activity functions in intact cells, but its membrane localization suggests a role in scavenging periplasmic H_2_O_2_ and protection against environmental H_2_O_2_. This peroxidase activity, however, had negligible effect in our experiments, because deletion of cytochrome *bd*-I abolished (rather than enhanced) PGRP-induced H_2_O_2_ production.

Cytochrome *bd*-I is important for bacterial growth and virulence, for example in *E. coli* urinary tract infections^33^ or colitis^25^. Cytochrome *bd*-I is also an attractive target for antibacterial agents, because it is only present in prokaryotes, but not in eukaryotic mitochondria. Cytochrome *bd*-I protects mycobacteria from killing by drugs targeting cytochrome c^34–37^, which is the opposite effect to the requirement for cytochrome *bd*-I in PGRP-induced killing reported in this study. However, similar to our results with PGRP in *E. coli*, combining cytochrome c inhibition with oxidative stress-inducing drug (clofazimine) in mycobacteria induces cytochrome *bd*-I-dependent enhancement of killing and production of reactive oxygen species^38^.

Some antibacterial peptides, namely aurachin D, gramicidin S, and microcin J25, selectively inhibit *E. coli* cytochrome *bd*-I redox activity^39–43^, and microcin J25 also induces cytochrome *bd*-I-dependent production of O_2_^–^^41–43^. However, these effects have been only shown in isolated membranes or proteins, but not in cells *in situ*, and the sensitivity to microcin J25-induced killing was similar in cytochrome *bd*-I- and *bo*_3_-deficient mutants. Also, gramicidin S and microcin J25 have additional targets in bacteria that are most likely responsible for their antibacterial effect: gramicidin S destroys cytoplasmic membrane integrity^40^ and microcin J25 inhibits RNA polymerase^41^, and, thus, their effects on cytochrome *bd*-I appear secondary. Human antibacterial peptide, LL-37, was also shown to induce a cytochrome *bd*-I-dependent burst of O_2_^−^ and HO^•^ production in *E. coli* cells before permeabilizing their cytoplasmic membranes, which is the main mechanism of LL-37-induced killing^44^. However, again, this O_2_^−^ and HO^•^ production did not significantly contribute to LL-37-induced killing, which was the same in cytochrome *bd*-I- and *bo*_3_-deficient mutants. Thus, these antibacterial peptides often have intracellular targets or permeabilize cytoplasmic membrane, their effects on cytochrome *bd*-I in cells *in situ* are negligible or unknown, and they are often transported into the periplasm and cytoplasm by specific transporters^41,43^. By contrast, PGRPs are large proteins, stay bound to the bacterial envelope, do not enter the cytoplasm^10^, do not permeabilize cytoplasmic membranes^4,6,10^, and induce large amounts of peroxide readily detectable in PGRP-treated cells^11,12^, which is dependent on cytochrome *bd*-I and correlates with the increased resistance of cytochrome *bd*-I-deficient mutants to PGRP-induced killing. Thus, the mechanisms of bacterial killing by small antibacterial peptides and PGRP are different.

PGRPs are conserved in evolution from insects to mammals and have retained their antimicrobial effectiveness for millions of years with no frequent emergence of fully resistant strains. The multiple factors responsible for this continued antimicrobial effectiveness of PGRPs include their ability to: (i) bind to multiple components of bacterial envelope (peptidoglycan, lipoteichoic acid, and LPS); (ii) simultaneously induce oxidative, thiol, and metal stress responses in bacteria, which individually are bacteriostatic, but in combination are bactericidal; (iii) induce oxidative, thiol, and metal stress responses in bacteria through three independent pathways; (iv) affect multiple metabolic pathways; and (v) synergize with other innate immune molecules, such as antimicrobial peptides (reviewed in^45^). Thus, emergence of PGRP resistance and disabling these multiple mechanisms would require simultaneous acquisition of resistance to these multiple separate antimicrobial mechanisms – a very low probability event. Our current results further elucidate this intricate ability of PGRPs to control bacterial growth and survival.

In conclusion, our results identify the sequence of events in PGRP-induced bacterial killing and uncover a bactericidal mechanism that can be exploited in the future for the development of new approaches to enhance resistance to infections and increase effectiveness of antibacterial therapy.

## Materials and Methods

### Materials

Human recombinant PGLYRP4 (used as a representative bactericidal PGRP) was expressed in S2 cells and purified as previously described^4,6^ in a buffer containing 10 mM TRIS (pH 7.6), with 150 mM NaCl, 10 µM ZnSO_4_, and 10% glycerol, and used at 100 µg/ml (0.87 µM, as PGLYRP4 is a 115 kDa disulfide-linked dimer^4^) final concentration, unless otherwise indicated. Paraquat (methyl viologen) was from Acros Organics and CCCP (carbonyl cyanide 3-chlorophenylhydrazone) from Molecular Probes (dissolved in DMSO, with equivalent amount of DMSO added to other groups). Fatty acids-free purified bovine serum albumin (BSA, used as a negative control) and other reagents were from Sigma-Aldrich, unless otherwise indicated.

### Bacteria, growth, and media

*E. coli* strains used in this study are listed in Supplementary Table S1^46^. Bacteria were grown aerobically overnight at 37 °C in an orbital shaker (250 rpm) in LB, diluted to OD_660_ = 0.02 into fresh LB and grown to OD_660_ = 0.6. Bacteria were centrifuged and suspended in the “Assay Medium”, which was the same for all the killing or metabolic experiments here and in our previous killing experiments on *E. coli*^6,10–12^. This Assay Medium contained (final concentrations): 5 mM TRIS (pH 7.6) with 2.5% glycerol, 150 mM NaCl, 5 µM ZnSO_4_, and 1% of 100% LB. *E. coli* MG1655 generation time in this assay medium at 37 °C was 30 min. The change in the medium from LB to the Assay Medium did not create any significant stress for bacteria^12^. Also, pre-incubation of cells in this medium for 30 min did not change their response to PGRP, as measured by respiration, extracellular acidification rate, H_2_O_2_ production, and killing^12^.

### Screening of Tn-mutant library

A highly saturated mini-Tn10 (Tn) insertion library in *E. coli* MG1655 containing ~200,000 unique mutants, generated and characterized previously^15^, was used in this study. Each mutant contained one transposon randomly inserted into the chromosome, with one insert per 20 bp on average and ~25 different transposon mutants for each gene/intergenic region, with nearly random distribution of insertions and the absence of large gaps or hot spots^15^. For each experiment, 5 µl of freshly thawed glycerol stock of the Tn library containing ~10^7^ CFU/5 µl was inoculated into 5 ml of LB and grown aerobically at 37 °C in an orbital shaker (250 rpm) for 3 generations. The bacteria were sedimented by centrifugation and suspended at ~2.5 × 10^6^ bacteria/ml in 50 µl of fresh warm Assay Medium with addition of 100 µg/ml PGRP, or 220 µg/ml PGRP, or 200 µg/ml BSA (control) and incubated at 37 °C aerobically with 250 rpm shaking for 3 hrs. These PGRP treatments resulted in ~50% and >99% reduction in CFU, respectively (higher concentration of PGRP than in other assays was required because of the high number of bacteria used in these experiments); BSA treatment resulted in ~40-fold increase in CFU in 3 hrs, compared with the initial inoculum. After 3-hr incubation each entire 50 µl culture was diluted with saline and plated on LB agar for single colonies on multiple 15 cm plates, which were then incubated at 37 °C aerobically overnight. Approximately 100,000, 50,000, and 1,000 colonies from BSA- and PGRP-treated groups, respectively, were then scraped from the plates and thoroughly mixed. The lower numbers of colonies in PGRP-treated groups represented all the surviving mutants (more resistant to PGRP), with ~50% and >99% of mutants (most sensitive to PGRP) killed by 100 and 220 µg/ml PGRP, respectively. For each treatment group, genomic DNA was extracted from 1-ml aliquot of pooled colonies containing ~5 × 10^9^ CFU using a Gentra Puregene Yeast/Bacteria Kit (Qiagen) and subjected to deep sequencing analysis. The entire experiment was repeated 3 times (3 biological replicates).

### Transposon sequencing (Tn-seq)

Illumina sequencing was carried out as described previously^15,47^. Briefly, genomic DNA was sonicated to produce 200- to 600-bp fragments. A poly(C) tail was added to DNA fragments by using terminal deoxynucleotidyl transferase. A first round of PCR with one transposon-specific primer (olj363) and one oligo (dG) primer (olj376) (Supplementary Table S2) was conducted to amplify the fragments. A second round of PCR with a nested primer (olj385) was conducted to add adaptors and bar codes for Illumina sequencing (with BSA, PGRP 50% survival, and PGRP <1% survival groups primers, bar codes are underlined in Supplementary Table S2). The sequencing was conducted on Illumina HiSeq 2000 at The Tufts University Core Facility (TUCF), Boston, MA, using olj386 custom sequencing primer and a standard Illumina index sequencing primer. The complete Tn-seq results have been deposited in NCBI SRA with accession number PRJNA549505 (samples SAMN12090400 - SAMN12090408) (https://www.ncbi.nlm.nih.gov/sra/PRJNA549505).

### Tn-seq data analysis

Read mapping and calculations were carried out on the Tufts University Galaxy server as described previously^15,47^. Briefly, Illumina reads were aligned to the *E. coli* MG1655 genome sequence using Bowtie^48^. The Tufts Galaxy server custom script was applied to enumerate read number for each insertion site. The insertion sites were then aggregated by annotated genes. In order to fully cover all genes and promoter regions, a GenBank file containing all intergenic regions was generated to allow insertion sites to aggregate to intergenic regions. The predicted number of reads for each gene was calculated based on the length of the gene and the total number of reads in the library. We identified 2,540 genes and 570 intergenic regions that contained 3 or more Tn insertion sites and we used these genes for further analysis, because >3 insertion sites are needed for reliable identification of mutants with changed frequency in the library^15^. For each gene in both BSA- and PGRP-treated groups we calculated Dval, which represents the predicted frequency of each gene in the library based on the length of the gene, the total length of the genome, and the total number of reads in the library; Dval = the number of actual reads for the gene/predicted number of reads for the gene. For each gene we then calculated the survival index, SI = Dval in PGRP-treated group/Dval in control BSA-treated group. The SI reflects a change in the frequency of each gene (Tn insertion mutant) following PGRP treatment. A neutral mutation with no effect on survival in the presence of PGRP has SI = 1, whereas SI > 1 denotes mutation that makes bacteria more resistant to PGRP, and SI < 1 denotes mutation that makes bacteria more sensitive to PGRP.

### Construction and verification of deletion mutants

We created gene deletion mutants (Supplementary Table S1) by replacing the entire coding sequence of the genes (in frame) with kanamycin resistance cassette using homologous recombination, as previously described^12,49^. We generated PCR fragments using primers (Supplementary Table S2) and individual mutants from the Keio collection^46^, digested the fragments with *Dpn*I, gel purified, and electroporated into *E. coli* MG1655 or BW25113 carrying the Red helper plasmid pKD46 (CGSC #7669). Transformed cells were recovered in SOC media with ampicillin and L-arabinose at 30 °C with shaking overnight, and kanamycin (30 µg/ml) resistant colonies were picked and verified by colony PCR with kanamycin-specific and gene-specific primers (Supplementary Table S2). The gene deletions in each mutant were then further verified by capillary sequencing using gene-specific primers. To create double deletion mutants, the kanamycin resistance gene was first removed from a single mutant using pCP20 plasmid. This plasmid has ampicillin resistance, shows temperature-sensitive replication, and thermal induction of Flp recombinase^50^. Mutants were transformed with pCP20, and ampicillin-resistant transformants were selected at 30 °C, further colony purified nonselectively at 43 °C, and then tested for loss of ampicillin as well as kanamycin resistance. Then, the second mutation was introduced by transforming pKD46 into the mutant whose kanamycin cassette had been removed, followed by the gene disruption protocol described above for generation of single deletion mutants.

### PGRP killing assay

*E. coli* were grown as described above in “Bacteria, growth, and media” section and suspended at 0.25 × 10^6^ bacteria/ml in 50 µl of fresh warm Assay Medium with addition of BSA or PGRP (100 µg/ml, except for Fig. 4a, where 50 µg/ml was used) and incubated at 37 °C aerobically with 250 rpm shaking for 3 hrs. The numbers of bacteria were then determined by colony counts^10–12^.

### H_2_O_2_ assay

H_2_O_2_ production was measured as previously described^12^ using Amplex Red Hydrogen Peroxide/Peroxidase Assay Kit (InVitrogen/Molecular Probes), based on enzymatic detection of H_2_O_2_ by horseradish peroxidase. *E. coli* were grown and suspended in 50 µl of Assay Medium at OD_660_ = 0.15 (~50 × 10^6^ bacteria/ml) as described above in “Bacteria, growth, and media” section, with addition of BSA (100 µg/ml), PGRP (100 µg/ml), paraquat (100 µM), CCCP (20 µM), or KCN (100 µM). Bacteria were then incubated aerobically at 37 °C on a shaker for 15 min (unless otherwise indicated). The reactions were then stopped by lysing bacteria with 40 mM HEPES, 4 mM EDTA, 400 mM KCl, 0.2% Triton X-100, pH 8.1 in a bath sonicator on ice for 5 min, and total amount of H_2_O_2_ was determined by measuring fluorescence^12^.

### Quinone assay

Quinones were extracted as described by Bekker *et al*.^22,51^. *E. coli* were grown as described above in “Bacteria, growth, and media” section and suspended at ~85 × 10^6^ bacteria/ml in 2.5 ml of fresh warm Assay Medium with addition of 300 µg/ml BSA or PGRP and incubated in 50-ml tubes at 37 °C aerobically with 250 rpm shaking for 15 min (higher concentration of PGRP than in other assays was required because of the high number of bacteria used in these experiments, which was needed to obtain sufficient amounts of quinones). Samples were immediately quenched with 7.5 ml of ice-cold methanol, immediately followed by addition of 7.5 ml of petroleum ether at 40–60 °C and vortexing for 1 min. The samples were centrifuged for 2 min at 900 *g* at 4 °C and the upper petroleum ether phase was transferred to a glass test tube and evaporated to dryness under a flow of nitrogen. The dried sample was re-extracted with another 7.5 ml of petroleum ether and evaporated to dryness under a flow of nitrogen as above, sealed in a nitrogen-purged glass tube, placed at −80 °C in the dark, and analyzed within 48 hrs. The extracted dried samples were reconstituted in 50 µL of 100% methanol just prior to the analysis, and oxidized and reduced quinones were quantified by LC/MS/MS as previously described^52^ at the Purdue University Bindley Bioscience Center Core Facility, West Lafayette, IN. Oxidized ubiquinone 9 (UQ_9_) and menaquinone 7 (MK_7_) were purchased from Cayman Chemicals and used as standards. Stock solutions were prepared in methanol and stored at −20 °C in the dark. Reduced forms were prepared daily by reduction with sodium borohydride and extraction with hexane, followed by reconstitution with methanol according to Ruiz-Jiménez *et al*.^52^.

An Agilent 6460 QQQ coupled to an Agilent 1200 Rapid Res LC system was used for the analysis. Water’s Xterra C18 2.1 × 100 mm, 3.5 µm column was used for the LC separation. The LC buffers were: (A) methanol:water:formic acid (74:25:1) with 5 mM ammonium formate and (B) methanol:formic acid (99:1) with 5 mM ammonium formate. The linear gradient was as follows: time 0 mins, 0% B; time 5 minutes, 100% B; time 15 minutes, 100% B; time 16 minutes, 0% B; time 20 minutes, 0% B. The auto sampler was set to 4 °C and in total darkness during the instrument acquisition. The data were acquired in multiple reaction monitoring mode (MRM). The source was set to a gas temperature of 330 °C, gas flow of 9 L/min, nebulizer pressure of 40 psi, sheath gas temperature of 250 °C, sheath gas flow of 7 L/min, the capillary was positive 4000 V, nozzle 1000 V, and ΔEMV of +400 V. Agilent Masshunter Quantitative analysis (v6.0) was used for the data analysis. *E. coli* UQ_8_, UQ_8_-H_2_, UQ_9_, UQ_9_-H_2_, and MK_8_ were detected and quantified based on UQ_9_, UQ_9_-H_2_, MK_7_, and MK_7_-H_2_ standards, consistent with the previously reported predominant synthesis of quinones with 8 and minor synthesis of quinones with 9 isoprenoid subunits in *E. coli*^20,22^.

### Membrane depolarization

Membrane depolarization was measured by flow cytometry with the membrane potential sensitive fluorescent probe DiOC_2_(3) (3,3-diethyloxacarbocyanine iodide) using Bac*Light*™ Bacterial Membrane Potential Kit (from Molecular Probes, ThermoFisher Scientific B34950)^53^ as recommended by the manufacturer. DiOC_2_(3) accumulates in the cell membrane and changes fluorescence from green to red at high membrane potential, and thus membrane depolarization is reflected by a decrease in red fluorescence. *E. coli* MG1655 were grown and suspended in 50 µl of Assay Medium at OD_660_ = 0.02 as described above in “Bacteria, growth, and media” section. DiOC_2_(3) (30 µM) and BSA (100 µg/ml), PGRP (100 µg/ml), CCCP (20 µM, concentration that caused complete membrane depolarization), or KCN (100 µM) were added, cultures were incubated aerobically at 37 °C on a shaker for 15 min, and fluorescence of ~5 × 10^5^ bacteria/culture was immediately measured by flow cytometry using MACSQuant (Miltenyi) flow cytometer with FITC (green) and PE (red) excitation and emission settings for green and red DiOC_2_(3) fluorescence. The extent of membrane depolarization was expressed as the ratios of mean red fluorescence intensity in BSA/treated cells ± SEM, with representative dot plots of green and red fluorescence intensity also shown.

### Gene expression arrays

Preparation of the whole genome expression arrays was described previously^11^ and the entire data for all the arrays were deposited in NCBI GEO with accession number GSE44211 (http://www.ncbi.nlm.nih.gov/geo/query/acc.cgi?acc=GSE44211). Here, from these arrays, we present and analyze for albumin- and PGRP-treated *E. coli* the expression of genes for formate dehydrogenases, proteins required for the assembly and activity of formate dehydrogenases, formate synthesis and transport, quinone synthesis, and cytochromes, which are the subject of this study and were not analyzed or presented previously. Briefly, exponentially growing *E. coli* MG1655 were suspended in the Assay Medium at OD_660_ = 0.3 as in “Bacteria, growth, and media” section, and incubated aerobically at 37 °C with 100 µg/ml albumin (control) or PGRP (human PGLYRP4) with 250 rpm shaking. RNA was then extracted using Ambion RiboPure-bacteria RNA extraction kit, cDNA was synthesized with random hexamer primers, fragmented, labeled with terminal transferase and biotin, and hybridized to whole genome Affymetrix *E. coli* Genome 2.0 Array GPL3154 using Affymetrix Hybridization Oven 640 and Affymetrix GeneChip Fluidics Station 450 and protocols provided by Affymetrix GeneChip Technical Manual. Scanning and data extraction were done using Affymetrix GeneChip Scanner 3000 and protocols provided by Affymetrix GeneChip Technical Manual. cDNA synthesis, labeling, hybridization, and scanning were performed at the Genomic and RNA Profiling Core facility, Baylor College of Medicine, Houston, TX. The entire experiment was repeated 3 times. Hybridization intensity data signals were normalized and analyzed as described^11^ using Affymetrix GeneChip Command Console Software. Signal intensities from 3 experiments (3 biological replicates) were used to calculate geometric means ± SEM. Transformed Ln(signal intensity) values were used for direct statistical comparisons of expression signals between PGRP-treated and control (albumin) groups.

### Annotation of gene functions

The functions of genes were annotated using the following web databases: EcoCyc: https://ecocyc.org/^17^ and RegulonDB: http://regulondb.ccg.unam.mx/index.jsp.

### Statistical analyses

Quantitative results are presented as arithmetic or geometric means ± SEM, with statistical significance of the differences between groups determined by the two-sample two-tailed Student’s *t*-test or paired Student’s *t*-test using Microsoft Excel; *P* ≤ 0.05 was considered significant. The *n* and *P* values are indicated in the figures.

## Supplementary information

Supplementary Information.

## Data Availability

All data generated or analyzed during this study are included in this published article (and its Supplementary Information) or have been deposited in NCBI as cited in the text under the accession numbers PRJNA549505 and GSE44211.
