# Supplementary material for: Formate dehydrogenase, ubiquinone, and cytochrome bd-I are required for peptidoglycan recognition protein-induced oxidative stress and killing in Escherichia coli
Source: Sci Rep. 2020 Feb 6;10:1993. doi: 10.1038/s41598-020-58302-1 (PMC7005000; doi:10.1038/s41598-020-58302-1)
Supplement: Supplementary file 1 — Supplementary Information. [file 41598_2020_58302_MOESM1_ESM.pdf]

Supplementary Information

Formate dehydrogenase, ubiquinone, and cytochrome *bd*-I are required for peptidoglycan recognition protein-induced oxidative stress and killing in *Escherichia coli*

Des R. Kashyap, Dominik A. Kowalczyk, Yue Shan, Chun-Kai Yang, Dipika Gupta & Roman Dziarski

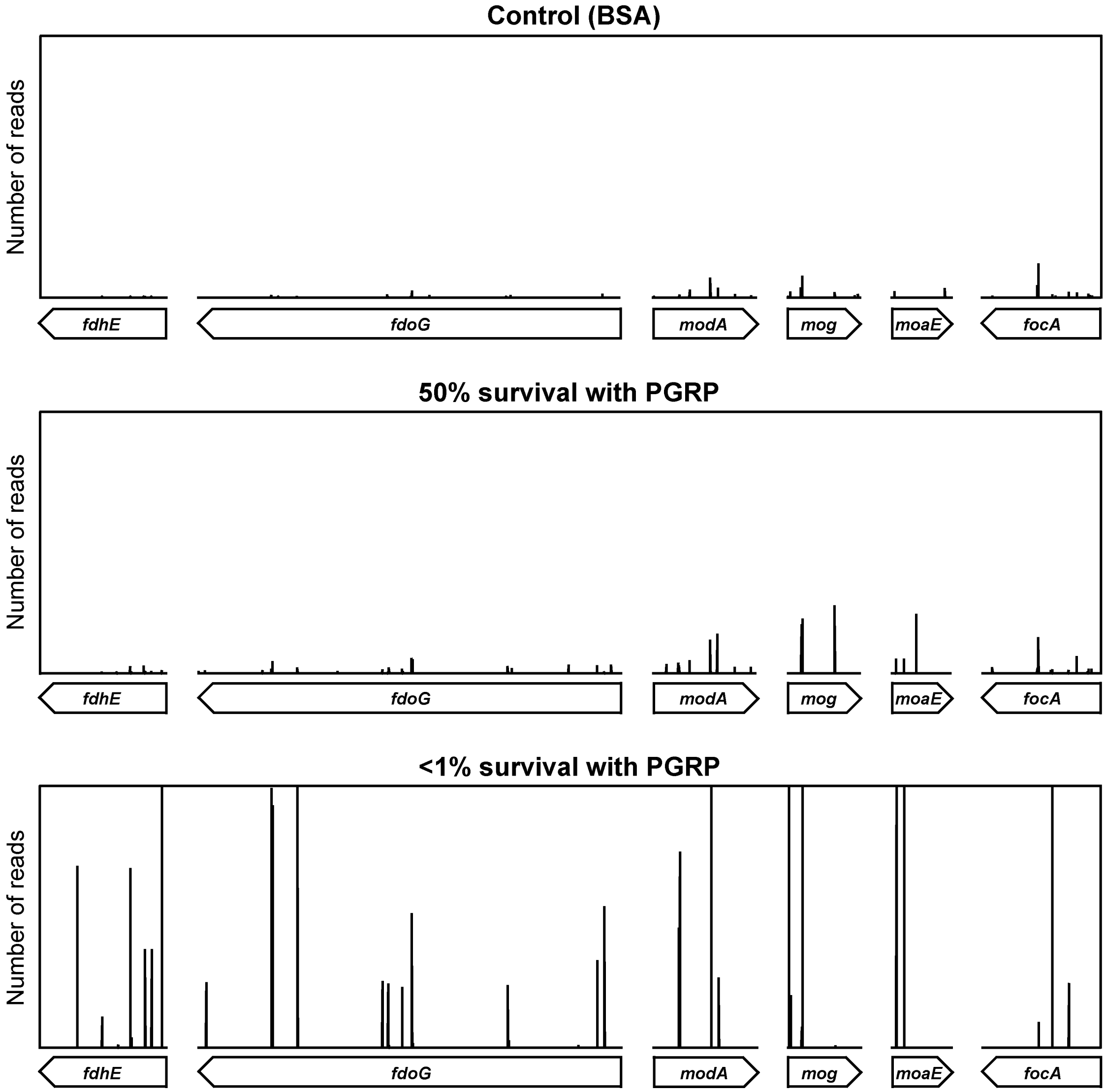

Supplementary Fig. S1. Tn insertion sites and the numbers of reads for FDH genes from Fig. 1 with the highest survival index in PGRP-treated cultures. *E. coli* Tn*10* insertion library was treated for 3 hrs with BSA as control or with PGRP at bactericidal (<1% survival) or sub-bactericidal (50% survival) concentration, and the numbers of reads for each Tn-insertion site in each gene was determined by Tn-seq. The height of each vertical line represents geometric mean of the number of sequencing reads at this position from 3 independent experiments (biological replicates). The complete Tn-seq results have been deposited in NCBI SRA with accession number PRJNA549505 (https://www.ncbi.nlm.nih.gov/sra/PRJNA549505).

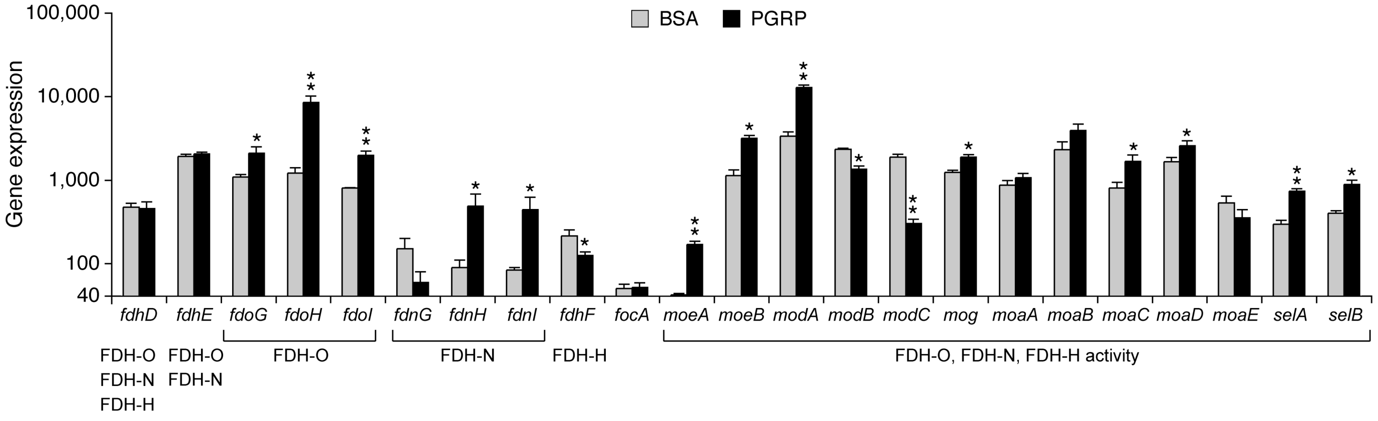

Supplementary Fig. S2. Expression of FDH and other genes required for the assembly and activity of FDH in PGRP-treated *E. coli*. *E. coli* MG1655 was treated with BSA or PGRP for 30 min and expression of the indicated genes was determined by whole genome expression arrays (NCBI GEO GSE44211). The results are geometric means ± SEM of signal intensities from 3 independent experiments (biological replicates); Y axis starts at the detection limit; * $P < 0.05$, ** $P < 0.001$ PGRP *vs* BSA.

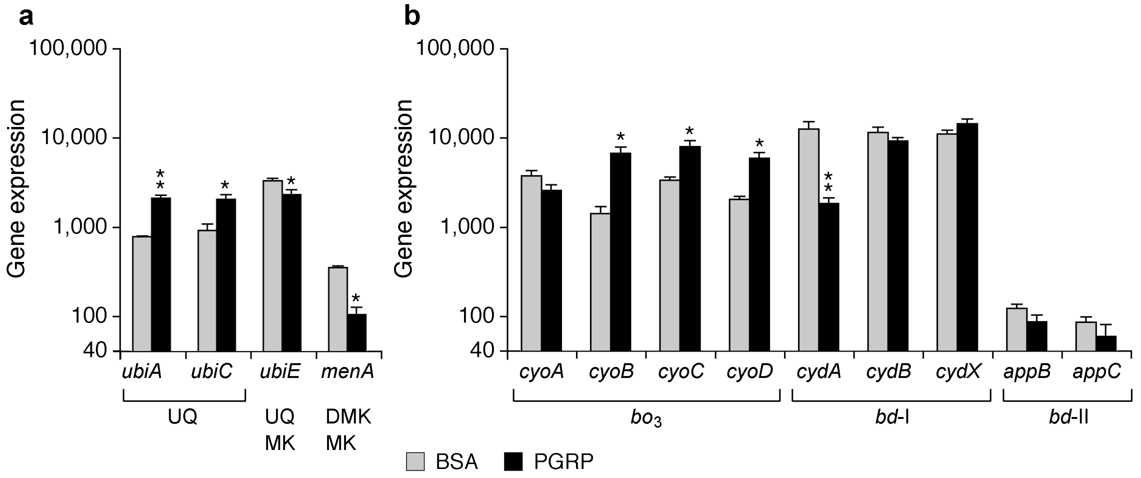

Supplementary Fig. S3. Expression of quinone synthesis and cytochrome genes in PGRP-treated *E. coli*. *E. coli* MG1655 was treated with BSA or PGRP for 30 min and expression of the indicated quinone synthesis genes (a) and cytochrome genes (b) was determined by whole genome expression arrays (NCBI GEO GSE44211). The results are geometric means ± SEM of signal intensities from 3 independent experiments (biological replicates); Y axis starts at the detection limit; * $P < 0.05$, ** $P < 0.001$ PGRP *vs* BSA.

Supplementary Table S1. Bacterial strains used in this study.

E. coli strain	Relevant genotype	Source or reference
MG1655	Parent K-12 strain for mutants, F-,λ-, *ilvG*-, rfb-50, *rph1*	ATCC 700926
DRK109 (Δ*ubiCA*)	MG1655::*ΔubiCA::kan*; Km^r	This Study
DRK110 (Δ*ubiE*)	MG1655::*ΔubiE::kan*; Km^r	This Study
DRK111 (Δ*menA*)	MG1655::*ΔmenA::kan*; Km^r	This Study
DRK112 (Δ*appB*)	MG1655::*ΔappB::kan*; Km^r	This Study
DRK113 (Δ*cyoB*)	MG1655::*ΔcyoB::kan*; Km^r	This Study
DRK114 (Δ*cydB*)	MG1655::*ΔcydB::kan*; Km^r	This Study
DRK117 (Δ*cyoB*Δ*appB*)	MG1655::*ΔcyoB::ΔappB::kan*; Km^r	This Study
DRK118 (Δ*cyoB*Δ*cydB*)	MG1655::*ΔcyoB::ΔcydB::kan*; Km^r	This Study
DRK121 (Δ*fdhE*)	MG1655::*ΔfdhE::kan*; Km^r	This Study
DRK122 (Δ*fdnG*)	MG1655::*ΔfdnG::kan*; Km^r	This Study
DRK123 (Δ*fdhD*)	MG1655::*ΔfdhD::kan*; Km^r	This Study
DRK124 (Δ*selB*)	MG1655::*ΔselB::kan*; Km^r	This Study
DRK125 (Δ*mog*)	MG1655::*Δmog::kan*; Km^r	This Study
DRK127 (Δ*fdoG*)	MG1655::*ΔfdoG::kan*; Km^r	This Study
DRK128 (Δ*fdhF*)	MG1655::*ΔfdhF::kan*; Km^r	This Study
DRK129 (Δ*focA*)	MG1655::*ΔfocA::kan*; Km^r	This Study
DRK130 (Δ*moeA*)	MG1655::*ΔmoeA::kan*; Km^r	This Study
DRK131 (Δ*modA*)	MG1655::*ΔmodA::kan*; Km^r	This Study
DRK141 (Δ*moaE*)	MG1655::*ΔmoaE::kan*; Km^r	This Study
DRK147 (Δ*ubiCA*)	BW25113::*ΔubiCA::kan*; Km^r	This Study
DRK148 (Δ*menA*)	BW25113::*ΔmenA::kan*; Km^r	This Study
BW25113	Parent K-12 strain for Keio collection mutants, *rrnB, DElacZ4787, HsdR514, DE(araBAD)567, DE(rhaBAD)568, rph-1*	Keio collection, National BioResource Project, National Institute of Genetics, Japan [46]
JW3866 (Δ*fdhD*)	BW25113 F-, *Δ(araD-araB)567*, *ΔlacZ4787*(::rrnB-3), λ^-, *rph-1*, *ΔfdhD758::kan*, *Δ(rhaD-rhaB)568*, *hsdR514*	
JW3862 (Δ*fdhE*)	BW25113 F-, *Δ(araD-araB)567*, *ΔlacZ4787*(::rrnB-3), λ^-, *rph-1*, *ΔfdhE754::kan*, *Δ(rhaD-rhaB)568*, *hsdR514*	
JW4040 (Δ*fdhF*)	BW25113 F-, *Δ(araD-araB)567*, *ΔlacZ4787*(::rrnB-3), λ^-, *rph-1*, *Δ(rhaD-rhaB)568*, *ΔfdhF774::kan*, *hsdR514*	
JW3865 (Δ*fdoG*)	BW25113 F-, *Δ(araD-araB)567*, *ΔlacZ4787*(::rrnB-3), λ^-, *rph-1*, *ΔfdoG757::kan*, *Δ(rhaD-rhaB)568*, *hsdR514*	
JW1470 (Δ*fdnG*)	BW25113 F-, *Δ(araD-araB)567*, *ΔlacZ4787*(::rrnB-3), λ^-, *ΔfdnG767::kan*, *rph-1*, *Δ(rhaD-rhaB)568*, *hsdR514*	
JW0887 (Δ*focA*)	BW25113 F-, *Δ(araD-araB)567*, *ΔlacZ4787*(::rrnB-3), λ^-, *ΔfocA728::kan*, *rph-1*, *Δ(rhaD-rhaB)568*, *hsdR514*	
JW0811 (Δ*moeA*)	BW25113 F-, *Δ(araD-araB)567, ΔlacZ4787*(::rrnB-3), *λ-, ΔmoeA727::kan, rph-1, Δ(rhaD-rhaB)568, hsdR514*	
JW0746 (Δ*modA*)	BW25113 F-, *Δ(araD-araB)567, ΔlacZ4787*(::rrnB-3), *ΔmodA735::kan, λ-, rph-1, Δ(rhaD-rhaB)568, hsdR514*	
JW0008 (Δ*mog*)	BW25113 F-, *Δmog-729::kan, Δ(araD-araB)567*, *ΔlacZ4787* (::rrnB-3), *λ-, rph-1, Δ(rhaD-rhaB)568, hsdR514*	
JW0768 (Δ*moaE*)	BW25113 F-, *Δ(araD-araB)567*, *ΔlacZ4787*(::rrnB-3), λ^-, *ΔmoaE757::kan*, *rph-1*, *Δ(rhaD-rhaB)568*, *hsdR514*	
JW3563 (Δ*selB*)	BW25113 F-, *Δ(araD-araB)567, ΔlacZ4787*(::rrnB-3), *λ-, ΔselB774::kan, rph-1, Δ(rhaD-rhaB)568, hsdR514*	
JW5581 (Δ*ubiE*)	BW25113 F-, *Δ(araD-araB)567*, *ΔlacZ4787*(::rrnB-3), λ^-, *rph-1*, *ΔubiE778::kan*, *Δ(rhaD-rhaB)568*, *hsdR514*	
JW0421 (Δ*cyoB*)	BW25113 F-, *Δ(araD-araB)567*, *ΔlacZ4787*(::rrnB-3), *ΔcyoB788::kan*, λ^-, *rph-1*, *Δ(rhaD-rhaB)568*, *hsdR514*	
JW0723 (Δ*cydB*)	BW25113 F-, *Δ(araD-araB)567*, *ΔlacZ4787*(::rrnB-3), *ΔcydB782::kan*, λ^-, *rph-1*, *Δ(rhaD-rhaB)568*, *hsdR514*	
JW0961 (Δ*appB*)	BW25113 F-, *Δ(araD-araB)567*, *ΔlacZ4787*(::rrnB-3), λ^-, *ΔappB722::kan*, *rph-1*, *Δ(rhaD-rhaB)568*, *hsdR514*	

Supplementary Table S2. Primers used in this study.

**Primers for Tn-seq**	
olj363	GTGTGGGCACTCGACATATGACAAG
olj376	GTGACTGGAGTTCAGACGTGTGCTCTTCCGATCTGGGGGGGGGGGGGGGG
olj385	AATGATACGGCGACCACCGAGATCTACACTCTTTGGGGGCCAAAATCATTAGGGGATTCATCAG
BSA<br>bar code	CAAGCAGAAGACGGCATACGAGATATCACGGTGACTGGAGTTCAGACGTGTGCTCTTCCGATCT
PGRP 50% survival<br>bar code	CAAGCAGAAGACGGCATACGAGATCGATGTGTGACTGGAGTTCAGACGTGTGCTCTTCCGATCT
PGRP <1% survival<br>bar code	CAAGCAGAAGACGGCATACGAGATTTAGGCGTGACTGGAGTTCAGACGTGTGCTCTTCCGATCT
olj386	ACACTCTTTGGGGGCCAAAATCATTAGGGGATTCATCAG
**Primers for extensions for gene deletions**	
ubiCA	ubiCA(H1) 5'-CTTTTCTGCGATACAATGCCTTTACGTTATGTAACGGAGAGTTCGGCATG-3'<br>ubiCA(H2) 5'-CCGGATGATCATCCGGCTTTTTACATCATCAGAAATGCCAGTAACTCAT-3'
ubiE	ubiE (H1) 5'-CAATCTGTTACACTTCTGGAACAATTTTTTGATGAGCAGGCATTGAGATG-3'<br>ubiE (H2) 5'-AAGGTTTAAAAGGCATTTCCGGTCTCCTGTCAGAACTTATAACCACGATG-3'
menA	menA (H1) 5'-TCATTGTTTGATGGGGCTGAAAGGCCCCATTTTTATTGGCGCGTATTATG-3'<br>menA (H2) 5'-ATCATCAATTGTTAATTGATATTTGTCAGTTATGCTGCCCACTGGCTTAG-3'
appB	appB (H1) 5'-ATGCAGAGTGAACAACCGACGCAGCAACAGGGGTAAAGGAGAAAATCATG-3'<br>appB (H2) 5'-CAAAGTAAATACCACATTGTTTCTGCTCCTTAGTACAACTCGTTTTCGTT-3'
cyoB	cyoB (H1) 5'-TGAGCCACGCGGAATCCGCCCATTAAAGGGGTTGAGGAAGAATAAAGATG-3'<br>cyoB (H2) 5'-GCGCGTGGGCAGTCGCGTGCGTCAAAGTATCAGTTGCCATTTTTCAGCCC-3'
cydB	cydB (H1) 5'-AGTCTTCCACGACTACTCAGCCGGCACGCTAAGACAGGAGTCGTCAAATG-3'<br>cydB (H2) 5'-TGCGAAATACCACATTTTTAGCTCCTTACTTAGTACAGAGAGTGGGTGTT-3'
fdhE	fdhE (H1) 5'-CCGCGCTGGTACCGTGAGGTCCGCAAGACAACGGAAAAGAAAGCTGAATG-3'<br>fdhE (H2) 5'-GACCTGCGCCACCCGGAAAACGTCAGAGATTACTCTCCTTCACCCGGAAA-3'
fdnG	fdnG (H1) 5'-CTGTTAGTTTCCTCGCGCAGTAATACCCCTGAAAAAAGAGGAAAGCAATG-3'<br>fdnG (H2) 5'-TGCGTTTCCATAGCCATCTGTTCGCCCCCTTACGCCTTCTCGATGTTGAC-3'
fdhD	fdhD (H1) 5'-AGAATACCATAATGTTGGTGTGTGTGTTCTTATCTGGTTAAGAGAAAGTG-3'<br>fdhD (H2) 5'-TTTAATATATTTCGCAGGACTTATTTTAATTAATTGCTCAAACGCTGCGG-3'
selB	selB (H1) 5'-TTACGCTGCCTTGAAGATGAGCAACGGTTTTTGGAGATGTTGTTGAAATG-3'<br>selB (H2) 5'-TTTATTTTAAAACTTAATTAATCATTTCCTTATTTTTCCGGAAATAATAA-3'
mog	mog (H1) 5'-CAGTGTATCATTCTGTTTAACGAGACTGTTTAAACGGAAAAATCTTGATG-3'<br>mog (H2) 5'-TTGAGATCCCCCCGCTCGGGGGGATTTTTTATTCGCTAACGTCGCGTCT-3'
fdoG	fdoG (H1) 5'-AAGCGAATGTAAATGGGACGTGACAATGTCGAAACAAGGAGCAATCCATG-3'<br>fdoG (H2) 5'-TGAGATTGATAAGCCATATGTCGTCTCCGTTACACCTTTTCCACATTCAC-3'
fdhF	fdhF (H1) 5'-GTAGTCGAGAGCGCGTATGCGTGATTTGATTAACTGGAGCGAGACCGATG-3'<br>fdhF (H2) 5'-TCCGAAAGGAGGCTGTAGAAAGGACGGTATTACGCCAGTGCCGCTTCGCG-3'
focA	focA (H1) 5'-ATGCTTTGTTAGTATCTCGTCGCCGACTTAATAAAGAGAGAGTTAGTGTG-3'<br>focA (H2) 5'-TTTTTATTTACTGCGTACTTCGACAACCATTAATGGTGGTCGTTTTCACG-3'
moeA	moeA (H1) 5'-TCTGACATAATAGGCAAATTCGATTTTGCCTCCGCAGGAGTGTTTTCATG-3'<br>moeA (H2) 5'-CAGCATCTCCTGATCGCTGAGTTCCGCCATTACAGGCCTCCGAACAACGC-3'
modA	modA (H1) 5'-TAGTCGTTATATTGTCGCCTACATAACGTTACATTAAGGGGTTACCAATG-3'<br>modA (H2) 5'-TGCCTGCCATTCTGGATCGGTCAGTATCATTACTTGATTGTAAATCCGTA-3'
moaE	moaE (H1) 5'-TGACGGCGACGAAGTAGCTTTCTTCCCGCCGGTAACCGGAGGTTAAGATG-3'<br>moaE (H2) 5'-CAGCGGTGCCTTATCCGGCTAACAAAAAACTACCAGCGTTTTGCCGCCTG-3'
**Primers for verification of deletions by PCR and sequencing**	
Kanamycin-specific primers	Kan F 5'-GTG CCC TGA ATG AAC TGC AGG ACG A-3'<br>Kan R 5'-GTG CCC AGT CAT AGC CGA ATA GCC T-3'
Gene-specific primers	
ubiCA	ubiCA F 5'-GGG CAT CGG TAA AGC GTA AGG-3'<br>ubiCA R 5'-GCA TCA GGC AAC CCA GAA GAA A-3'
ubiE	ubiE F 5'-CCA TTG GGA GTA GTT AAG CCG-3'<br>ubiE R 5'-ACA GGA AGG TGT TGA GCA GAC-3'

menA	menA F 5'-TGG ATG CGT TGG TGG CAG ATG A-3'<br>menA R 5'-GGC TTA ACA TTC AGT TGC TGC G-3'
appB	appB F 5'-GCG ATA CAG GAC ATC TTA CCG A-3'<br>appB R 5'-TTG CCT GAT GCG ATG CTA ATG C-3'
cyoB	cyoB F 5'-GTG AAA CCA GAC TTG TTT GCC G-3'<br>cyoB R 5'-TTC ACC AGA ACG GCA TAG GTA GC-3'
cydB	cydB F 5'-CTC AAT GGT GCT GAT TTG CGG C-3'<br>cydB R 5'-GGT CCC AAA ACA TAC ATC CTG C-3'
fdhE	fdhE F 5'-GGT GGA AGG ATG GGT TAC CAG CG-3'<br>fdhE R 5'-CCT TAT TGA TGT GGT GGT GGG T-3'
fdnG	fdnG F 5'-CTG TCA GGC AGT GGT GCG TTT TT-3'<br>fdnG R 5'-TAC AGC CGA TAC AGG TGG AAA CG-3'
fdhD	fdhD F 5'-TTA CAT TCG CTT GCT GCG TGT GC-3'<br>fdhD R 5'-CGA GGG ACA TGA TGG CGA CTA TA-3'
selB	selB F 5'-TAG CCA CCT TGA GTC ATT AGC CGC-3'<br>selB R 5'-GAA AGT GTA AAA AAG GGC GGG CAG-3'
mog	mog F 5'-ATC ACT GAG TCC GAG TTC CTG TG-3'<br>mog R 5'-GCA ATC TAT CTG GCG ATG GGT GA-3'
fdoG	fdoG F 5'-GTT ACA AAC CCT TCC TGG ATG GAG-3'<br>fdoG R 5'-TGA GTT TCG CCA CTT CTT CCT GG-3'
fdhF	fdhF F 5'-ACT GAC GAC AGC CTG TTT TTC G-3'<br>fdhF R 5'-TCG TTG GCG TGG GCA AAG AAT GT-3'
focA	focA F 5'-GCC TAT AAG CCA GGC GAG ATA TGA-3'<br>focA R 5'-CTT CAT TCT GCC AGT CAC CTT TGG-3'
moeA	moeA F 5'-TTC GTC CAT TAA GGG TGG TTC GC-3'<br>moeA R 5'-CGA GCA GCG TCA GGT TAC CGA CA-3'
modA	modA F 5'-TTC CCT GAA TGC CCG CTT AGT CG-3'<br>modA R 5'-CAA CGC ACC AGT AAC CAG GCA AA-3'
moaE	moaE F 5'-GCA AAT TAC TGG CTG CCG TCA AC-3'<br>moaE R 5'-GCA GCG TAA ACG TAT GTA CTG AGC-3'
